# Prostaglandin E_2_ induces dendritic cell dysfunction in skin involvement of breast cancer

**DOI:** 10.1038/s41419-026-08519-1

**Published:** 2026-02-25

**Authors:** Jiawen Wang, Xiaoming Zhong, Xu Liu, Zhiyun Qian, Jingkun Zhu, Huayue Lin, Jiahui Zhang, Wei Zhang, Sicong Du, Linbin Yang, Man Nie

**Affiliations:** 1 Guangdong Provincial Key Laboratory of Malignant Tumor Epigenetics and Gene Regulation, Guangdong-Hong Kong Joint Laboratory of RNA Medicine, Sun Yat-sen Memorial Hospital, Sun Yat-sen University, Guangzhou, PR China; 2 Breast Tumor Center, Sun Yat-sen Memorial Hospital, Sun Yat-sen University, Guangzhou, PR China; 3 Department of Thoracic Surgery, Sun Yat-sen Memorial Hospital, Sun Yat-sen University, Guangzhou, PR China; 4 Department of Medical Oncology, State Key Laboratory of Oncology in South China, Guangdong Provincial Clinical Research Center for Cancer, Collaborative Innovation Center for Cancer Medicine, Sun Yat-sen University Cancer Center, Guangzhou, PR China

**Keywords:** Breast cancer, Conventional dendritic cells, Immunosuppression, Translational immunology, Translational research

## Abstract

The skin involvement (SI) of breast cancer exhibits suboptimal to standard treatment and poor prognosis. Dendritic cells (DCs) are essential to maintain immune homeostasis. However, the role of cutaneous DCs in skin lesions of breast cancer remains elusive, limiting the development of therapeutic approaches. Here, skin tissues from 47 breast cancer patients were analyzed for different immune cell infiltration, showing a significant reduction in DC number and activation in lesional skin. Transcriptome analyses, in vitro antigen processing and T lymphocyte priming assays of primary cutaneous DCs from breast cancer patients corroborated impaired antigen processing and T lymphocyte priming in lesional skin. Mechanistically, metabolomic analyses profiled the microenvironment of lesional and non-lesional skin and revealed increased prostaglandin E_2_ (PGE_2_) levels in the lesional skin, which could inhibit DC activation. Inhibiting PGE_2_ in vivo effectively restored the activation of DCs and CD8^+^ T lymphocytes and attenuated the skin involvement in mouse models of different cancer types. Clinically, the PGE_2_ levels were negatively correlated with DC infiltration in the skin of breast cancer patients, and low PGE_2_ expression and high DC activation were associated with better patient outcomes. Collectively, our study reveals that PGE_2_ induces DC dysfunction in the skin involvement of breast cancer, highlighting the potential of targeting PGE_2_ for managing patients with skin involvement.

**Figure:**
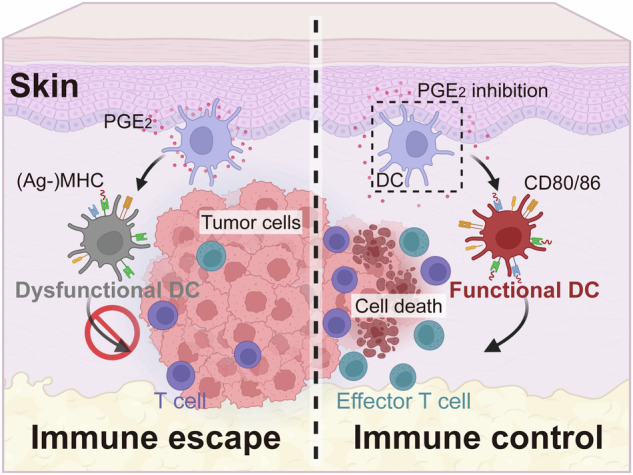
**A schematic diagram illustrating PGE**_**2**_ **induced DC dysfunction in the skin**. PGE_2_ induces DC dysfunction in the skin involvement of breast cancer, and PGE_2_ inhibition restores DC activation and relieves skin involvement. Ag, antigen; CD, cluster of differentiation; DC, dendritic cell; MHC, major histocompatibility complex; PGE_2_, prostaglandin E_2_.

**A schematic diagram illustrating PGE**_**2**_ **induced DC dysfunction in the skin**. PGE_2_ induces DC dysfunction in the skin involvement of breast cancer, and PGE_2_ inhibition restores DC activation and relieves skin involvement. Ag, antigen; CD, cluster of differentiation; DC, dendritic cell; MHC, major histocompatibility complex; PGE_2_, prostaglandin E_2_.

## Highlights

- Skin involvement in breast cancer is not uncommon with suboptimal treatment response
- DCs in lesional skin show compromised activation and functions
- PGE_2_ inhibits DC activation and negatively correlates with DC infiltration in the skin
- Modulating DC or targeting PGE_2_ holds great potential for skin involvement management

## Background

The occurrence of skin involvement (SI) in breast cancer is not uncommon, with the prevalence ranging from 5% to 20% [1–3]. Considering the high incidence of breast cancer, the number of breast cancer patients with skin involvement cannot be overlooked [4, 5]. The patients, however, showing a suboptimal response to current standard treatment, present an incidence rate up to 50% developing disease progression [6–8] and a mortality rate exceeding 30% within the first five years following diagnosis [8–11]. The immune microenvironment of lesional skin in breast cancer with skin involvement remains unknown and elucidating the interaction between tumors and immune cells holds the potential to facilitate the development of innovative therapies.

In the skin, tightly regulated multicellular networks are required to initiate protective innate and adaptive immune responses [12]. Innate immune cells, particularly dendritic cells (DCs), are essential for induction of adaptive immunity [13]. They sense the presence of pathogens through pattern recognition receptors (PRRs) and subsequently modulate the immune system to eliminate microbe in the course of skin infection [14, 15]. However, the activation of DCs ought to be well-balanced to maintain the immune homeostasis. In skin inflammable disorders like psoriasis and atopic dermatitis, the DCs exhibit aberrant activation and secrete multiple cytokines, thereby contributing to the dysregulated skin inflammation. Whereas in the context of malignancies, the functional suppression of DCs may serve as one of the strategies employed by cancer cells to evade immune surveillance [16, 17]. Currently, the role of DCs in breast cancer is mainly explored in primary tumors as well as lung, liver or bone metastases [16, 18, 19]. Further investigation is required to determine whether DCs also play a role in skin involvement and decipher the underlying mechanisms.

Here, the immune cell profiles in skin samples of breast cancer patients were analyzed by immunofluorescence, revealing that DCs in lesional skin were less activated than those in non-lesional skin. And the DCs were isolated from the fresh skin tissues of breast cancer patients with or without skin involvement, for high-throughput sequencing and in vitro experiments. These data further confirmed the impaired antigen processing and diminished T lymphocyte priming by DCs in lesional skin. Metabolomic analyses revealed elevated prostaglandin E_2_ (PGE_2_) levels in the lesional skin, which could facilitate the dysfunction of cutaneous DCs and targeting PGE_2_ in vivo effectively restored the activation of DC and CD8^+^ T lymphocytes and attenuated the cutaneous involvement of mouse breast cancer. Clinically, the infiltration of activated DCs was negatively correlated with PGE_2_ expression in skin samples and further influenced patient outcomes. Overall, our study provided novel insights into the characteristics of DCs in the skin of breast cancer patients, highlighting the detrimental role of PGE_2_ on DC function and immune surveillance. These findings provide a promising therapeutic approach of targeting PGE_2_ in breast cancer patients with skin involvement.

## Methods

### Patients and samples

Breast cancer patients who had undergone mastectomy or skin excisional biopsy at Sun Yat-sen Memorial Hospital between 2015 and 2024 were included in the study. The inclusion criteria were as follows: (1) pathologically diagnosed as breast cancer; (2) archived formalin-fixed paraffin-embedded (FFPE) or freshly excised skin samples were available for further analyses; (3) complete medical history record. All of the skin samples were further confirmed by experienced pathologists. Besides making FFPE slices, part of freshly excised skin tissues was used for in vitro assays. The treatment response of the patient was evaluated according to the Response Evaluation Criteria in Solid Tumors (RECIST, version 1.1). The informed consents were signed by all patients and the research protocol was approved by the internal review and ethics board of Sun Yat-sen Memorial Hospital, Sun Yat-sen University.

### Cell culture

The mouse breast cancer cell line EO771 and the melanoma cell line B16F10 were obtained from CH3 Biosystems and Cell Bank/Stem Cell Bank, Chinese Academy of Sciences, respectively. All the cells were cultured in a humidified 37 °C incubator with 5% CO_2_ and grown in RPMI 1640 or DMEM medium with 10% fetal bovine serum (FBS, all from Thermo Fisher Scientific).

### Animal experiments

Wild-type 6- to 12-week-old female C57BL/6J and BALB/c mice were purchased from Guangdong GemPharmatech and fed in specifical pathogen-free (SPF) environment in the Animal Experiment Center, Sun Yat-sen University. For experimental skin involvement of breast cancer and melanoma, 5 × 10^6^ EO771 cells and 1 × 10^5^ B16F10 cells were resuspended in 100 μL sterile and endotoxin-free PBS and subcutaneously (s.c.) injected into the flanks of C57BL/6J mice, respectively [20, 21]. For PGE_2_ antagonism [22, 23] or cyclooxygenase (COX) inhibition [24], 10 mg/kg PF-04418948 and L-161,982 (prostaglandin E_2_ receptor [EP] subtype 2 and 4 antagonists, from Selleck and TargetMol, respectively), or 5 mg/kg celecoxib (a COX-specific inhibitor, Selleck), were sequentially dissolved in 5% dimethyl sulfoxide (DMSO, Sigma-Aldrich), 40% polyethylene glycol (PEG) 300, 5% Tween-80 (both from Selleck) and 50% H_2_O, and intra-peritoneally (i.p.) injected into mice daily, respectively. The animal experiment was approved by the Institutional Animal Care and Use Committee, Sun Yat-sen University.

### Statistical analysis

Student’s *t*-test or Mann-Whitney’s *U*-test was used to determine the significance between two groups. Analysis of variance (ANOVA) or Kruskal-Wallis’s *H*-test, and subsequent Dunnett’s multiple comparisons test were used to determine the significance among three or more groups. Chi-square test or Fisher’s precision probability test were applied for categorical variables. Correlation was determined using Spearman’s or Pearson’s correlation coefficient (*r*) and subsequent *t*-test. Overall survival (OS), relapse-free survival (RFS), disease-specific survival (DSS), or progression-free survival (PFS) were plotted using Kaplan-Meier method and the significance was determined using Gehan-Breslow-Wilcoxon test, log-rank test, or Cox proportional hazard regression model with the Enter method (for univariable analysis) or Stepwise Forward likelihood ratio (LR) method (for multivariable analysis). When the *P*-value < 0.05, the test was considered as significant, and all *P*-values corresponded to two-tailed significant tests. Statistical analyses were performed, and data graphs were plotted using Prism (version 9.3.1, GraphPad) or Statistical Product and Service Solutions Statistics (version 25, International Business Machines Corporation).

Other detailed methods are provided in Supplementary files.

## Results

### Lower infiltration and decreased activation of CD141^+^ DCs in lesional skin of breast cancer patients

**Fig. 1:**
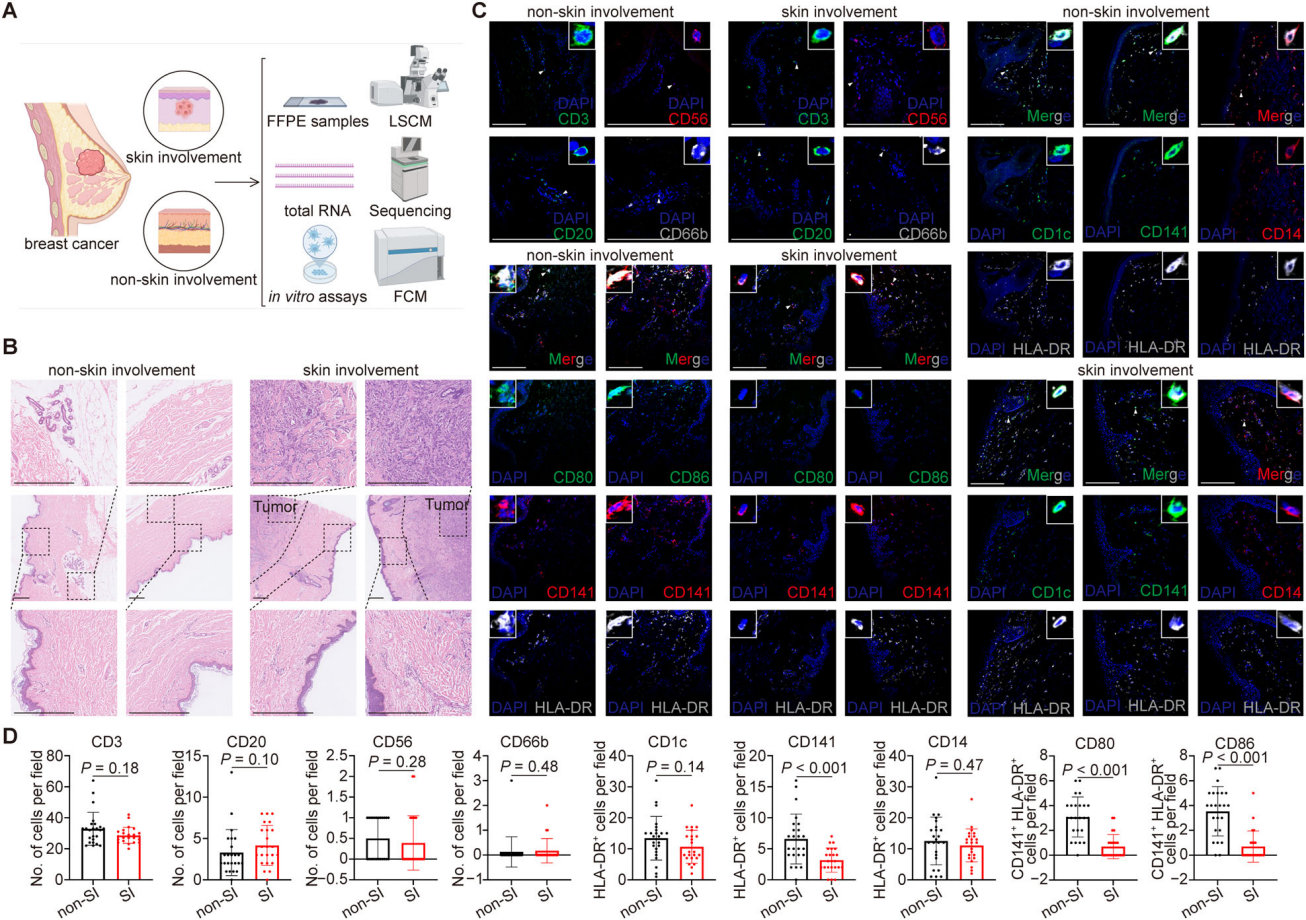
Different subsets of immune cells in breast cancer patients with or without skin involvement. **A** A schematic diagram illustrating patient samples detection. **B** Representative H&E images of breast cancer patients with or without skin involvement (SI). Black dashed lines showed approximate tumor margins; black dashed squares showed the magnified regions. Scale bar, 500 μm. **C**, **D** Representative immunofluorescent staining of T lymphocytes, B lymphocytes, NK cells, granulocytes and myeloid subsets, as well as DC activation in skin tissues of breast cancer patients with (*n* = 23) or without (*n* = 24) skin involvement. White triangles indicated the cells shown in the upper-right or upper-left insets in each image. Scale bar, 200 μm. Data were mean ± standard deviation (SD); significance was determined using a two-tailed Student’s *t*-test (CD1c and CD14 in **D**) or Mann-Whitney’s *U*-test (others in **D**). CD cluster of differentiation, DAPI 4’,6-diamidino-2-phenylindole, FCM flow cytometry, FFPE formalin-fixed paraffin-embedded, HLA human leukocyte antigen, LSCM laser-scanning confocal microscopy, non-SI breast cancer patients without skin involvement, SI breast cancer patients with skin involvement.

A total of 47 female breast cancer patients who had undergone breast surgery or skin excisional biopsy were included in this study (*n* = 23, with skin involvement; *n* = 24, without skin involvement) (Fig. 1A, B and Table S1). The median (range) age of patients was 49 (24‒77) years. Clinically, 21 of 23 (91%) patients with skin involvement presented with skin nodules and only 2 patients showed carcinoma erysipelatoides. Pathologically, there was no significance in the expression of estrogen receptor (ER; *P* = 0.19), progesterone receptor (PgR; *P* = 0.77), human epidermal growth factor receptor 2 (HER2; *P* > 0.99) and proliferative index Ki67 (*P* = 0.58) between these two groups.

The infiltration profiles of immune cells in breast cancer patients with or without skin involvement were detected by immunofluorescence. CD3, CD20, CD56 and CD66b were used to determine T lymphocytes, B lymphocytes, natural killer (NK) cells and granulocytes, respectively; CD1c, CD141 and CD14 combined with human leukocyte antigen (HLA)-DR were used to identify myeloid subsets [25, 26]. The densities of T lymphocytes (*P* = 0.18), B lymphocytes (*P* = 0.10), NK cells (*P* = 0.28) and granulocytes (*P* = 0.48) were similar between lesional and non-lesional skin. However, there was a significant reduction in the number of CD141^+^HLA-DR^+^ DCs (*P* < 0.001), rather than CD1c^+^HLA-DR^+^ cells (*P* = 0.14) or CD14^+^HLA-DR^+^ cells (*P* = 0.47) in the lesional skin. More importantly, these CD141^+^HLA-DR^+^ DCs exhibited lower expression levels of CD80/CD86 (both *P* < 0.001) (Fig. 1C, D), which are recognized as activation markers of DCs [27]. Collectively, the lesional skin of breast cancer patients exhibited reduced infiltration and decreased activation of CD141^+^ DCs.

### The antigen processing and priming abilities of DCs in lesional skin were compromised

**Fig. 2:**
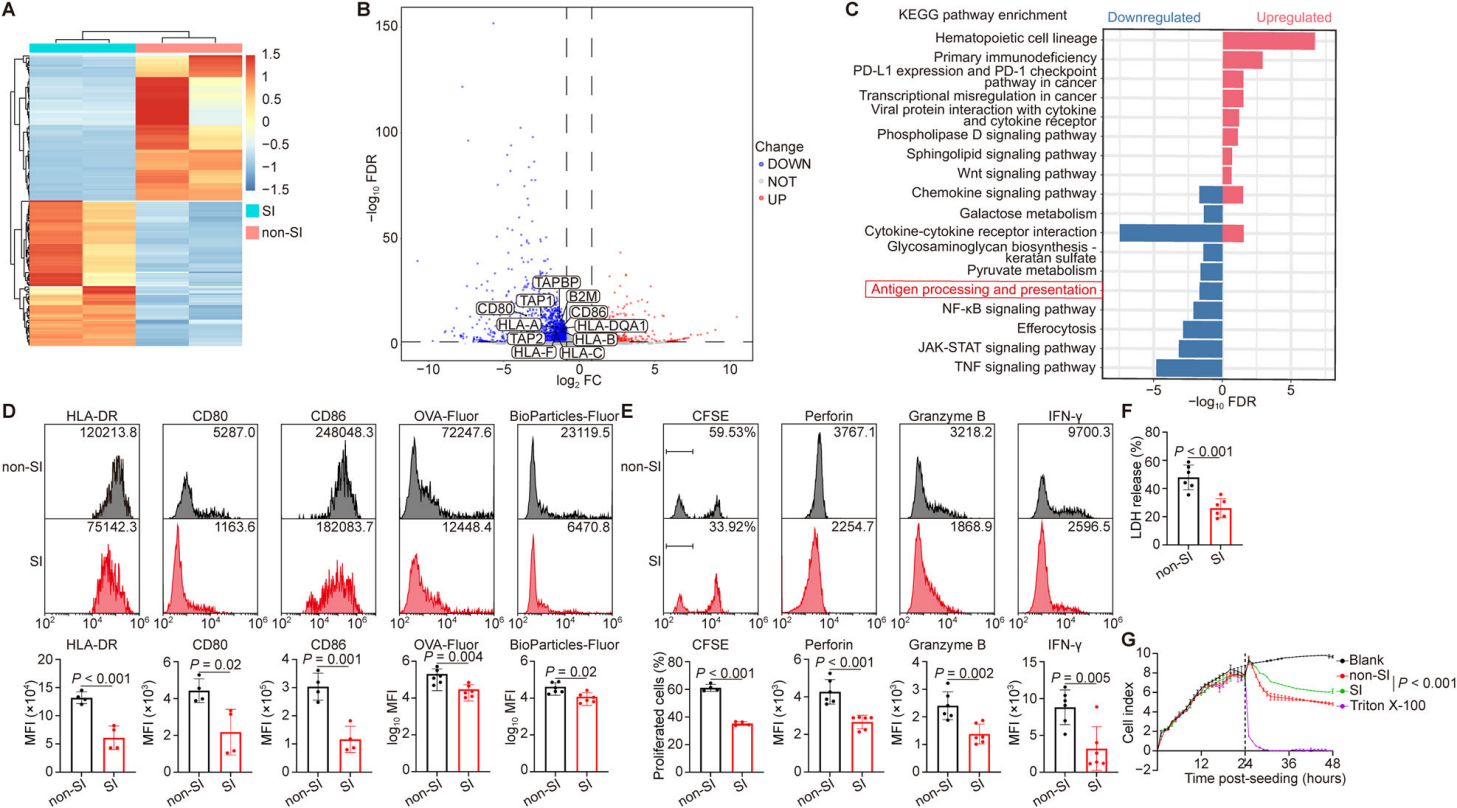
Antigen processing and priming of DCs in lesional and non-lesional skin of breast cancer patients. **A**–**C** Transcriptomic analyses of isolated DCs from breast cancer patients with or without skin involvement. *n* = 2 biological replicates per group. **A** Heatmap showed the gene expression levels and clusters. Light red colors indicated the higher mean expression levels while navy blue indicated the lower mean expression levels. **B** The volcano plot showed differentially expressed genes (DEGs). *CD80*, *CD86* and antigen processing related genes (*B2M*, *TAPBP*, *TAP1*, *TAP2*, *HLA-A*, *HLA-B*, *HLA-C*, *HLA-DQA1* and *HLA-F*) were highlighted. Light red colors indicated significantly higher mean expression levels, navy blue colors indicated significantly lower mean expression levels and gray colors indicated insignificant expression levels in lesional skin. **C** KEGG enriched pathways. Light red colors indicated the upregulated pathways while navy blue colors indicated the downregulated pathways in lesional skin. The red box marked the antigen processing and presentation pathway. **D**–**G** The DCs were isolated from the lesional and non-lesional skin tissues of breast cancer patients, applied to in vitro function assays and analyzed by flow cytometry or spectrophotometry. **D** Representative flow cytometry plots showed the expression of HLA-DR, CD80 and CD86 (*n* = 4 independent experiments), as well as the uptake and processing of fluorescence-conjugated OVA and bioparticles (*n* = 6 independent experiments) by DCs. **E** Representative flow cytometry plots showed the proliferation (*n* = 4 independent experiments) and expression of cytotoxic cytokines (*n* = 6 independent experiments) in T lymphocytes primed by DCs. **F**, **G** The cytotoxic effect of T lymphocytes primed by DCs on tumor cells was assessed using LDH release assay (**F**, *n* = 6 independent experiments) and real-time impedance monitoring assay (**G**, *n* = 3 biological replicates per group). The black dashed line indicated the time point at which T lymphocytes were added. Data were mean ± SD; significance was determined using a two-tailed Student’s *t*-test (HLA-DR, CD80 and CD86 in **D**, **E**, **F**) or Mann-Whitney’s *U*-test (others in **D**), or one-way analysis of variance (ANOVA) and subsequent Dunnett’s multiple comparisons test at the last time point (**G**). CD cluster of differentiation, CFSE 5(6)-carboxyfluorescein diacetate N-succinimidyl ester, FC fold change, FDR false discovery rate, Fluor fluorescence, HLA human leukocyte antigen, IFN interferon, KEGG Kyoto Encyclopedia Genes and Genomes, LDH lactate dehydrogenase, MFI mean fluorescence intensity, non-SI breast cancer patients without skin involvement, OVA ovalbumin, SI breast cancer patients with skin involvement.

To further explore the function of DCs in lesional skin, we performed transcriptome sequencing of DCs isolated from lesional and non-lesional skin of breast cancer patients (Fig. 2A). DCs from lesional skin displayed downregulation of antigen-processing genes, as well as *CD80* and *CD86* (Fig. 2B). Consistent with this, KEGG enrichment analysis revealed significant suppression of the antigen processing and presentation pathway in these DCs (Fig. 2C). To validate this phenomenon, we evaluated the expression of HLA-DR, CD80, and CD86, as well as the antigen processing capacity of cutaneous DCs from breast cancer patients using fluorescence-conjugated ovalbumin (OVA) and bioparticles [28, 29]. As expected, we observed significantly reduced expression of HLA-DR, CD80, and CD86, as well as lower uptake and processing in lesional skin DCs compared to non-lesional controls (Figs. 2D and S1A, B). Moreover, DCs isolated from lesional skin exhibited an impaired capacity to prime T lymphocytes, resulting in reduced T lymphocyte proliferation and cytotoxic cytokines expression (Figs. 2E and S1C). Consequently, T lymphocytes primed by lesional DCs showed diminished cytotoxicity, as measured by lactate dehydrogenase (LDH) release and real-time impedance assays (Fig. 2F, G) [30–32]. This dysfunctional phenotype was conserved in a breast cancer mouse model with skin involvement [20, 21], which displayed fewer DCs in lesions (Fig. S2A, B). These DCs exhibited significantly lower fluorescence intensity for MHC-Ⅱ, CD80, CD86, as well as diminished antigen-processing capacity (Figs. S2C, S3A), and primed T lymphocytes with lower proliferation and cytokine expression (Figs. S2D, E, S3B).

Collectively, our results indicate that DCs in lesional skin are functionally impaired in antigen processing and T lymphocyte priming.

### The accumulation of PGE_2_ hindered the activation and function of DCs in lesional skin

**Fig. 3:**
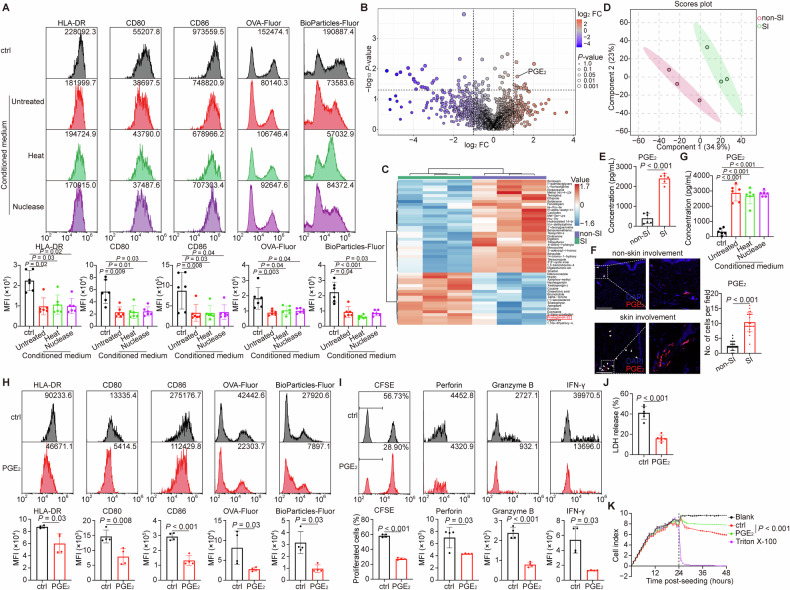
Increasing PGE_2_ in breast cancer with skin involvement impeded the activation and functions of DCs. **A** Cutaneous DCs derived from breast cancer patients were cultured in the absence (control) or presence of un-, heat- or nuclease-treated conditioned medium (CM), and analyzed by flow cytometry. Representative flow cytometry plots showed the expression of HLA-DR, CD80 and CD86, as well as the uptake and processing of fluorescence-conjugated OVA and bioparticles by DCs. *n* = 6 independent experiments. **B**–**D** Metabolomic analyses of skin involvement tissues from the experimental mouse model. *n* = 3 biological replicates per group. **B** The volcano plot showed differentially expressed metabolites. PGE_2_ was marked. Red colors indicated the higher mean expression levels while blue indicated the lower mean expression levels. **C** Heatmap visualization was utilized to identify metabolites that exhibited significantly higher (redder) or lower (bluer) levels between the two groups. The top 50 metabolites were shown according to *P*-values and each colored cell on the map corresponded to the relative concentration value of metabolites. The red box marked PGE_2_. **D** The scores plot by partial least squares discriminant analysis (PLS-DA) showed a clear separation between the two groups. **E** The PGE_2_ concentration in lysates of skin involvement tissues from breast cancer patients was measured by ELISA. *n* = 6 per group. **F** Representative immunofluorescent staining of PGE_2_ in skin tissues of breast cancer patients with (*n* = 23) or without (*n* = 24) skin involvement. White triangles indicated PGE_2_; white dashed squares showed the magnified regions. Scale bar, 200 μm. **G** The PGE_2_ concentration in the supernatant of control or indicated CM was measured by ELISA. *n* = 6 independent experiments. **H**–**K** Cutaneous DCs derived from breast cancer patients were treated with either the control or PGE_2_, applied to in vitro function assays and analyzed by flow cytometry or spectrophotometry. *n* = 4 independent experiments (**H**, **I**), 6 independent experiments (**J**) or 3 biological replicates per group (**K**). **H** Representative flow cytometry plots showed the expression of HLA-DR, CD80 and CD86, as well as the uptake and processing of fluorescence-conjugated OVA and bioparticles by DCs. **I** Representative flow cytometry plots showed the proliferation and expression of cytotoxic cytokines in T lymphocytes primed by DCs. **J**, **K** The cytotoxic effect of T lymphocytes primed by DCs on tumor cells was assessed using LDH release assay (**J**) and real-time impedance monitoring assay (**K**). The black dashed line indicated the time point at which T lymphocytes were added. Data were mean ± SD; significance was determined using a two-tailed Kruskal-Wallis’s *H*-test and subsequent Dunnett’s multiple comparisons test (**A**), Student’s *t*-test (**E**; CD80 and CD86 in **H**; CFSE and Granzyme B in **I**; **J**), Mann-Whitney’s *U*-test (others in **F**, **H** and **I**), or one-way analysis of variance (ANOVA) and subsequent Dunnett’s multiple comparisons test (**G**; at the last time point in **K**). CD cluster of differentiation, CFSE 5(6)-carboxyfluorescein diacetate N-succinimidyl ester, ctrl control, DAPI 4’,6-diamidino-2-phenylindole, FC fold change, Fluor fluorescence, HLA human leukocyte antigen, IFN interferon, LDH lactate dehydrogenase, MFI mean fluorescence intensity, non-SI breast cancer patients (or mice bearing breast cancer) without skin involvement, OVA ovalbumin, PGE_2_ prostaglandin E_2_, SI breast cancer patients (or mice bearing breast cancer) with skin involvement.

To investigate the mechanisms underlying DC dysfunction in lesional skin, we treated cutaneous DCs from breast cancer patients with conditioned medium (CM) from breast cancer cells. This treatment markedly impaired DC activation and antigen-processing capacity. The suppressive effect of tumor CM persisted despite heat treatment (to denature proteins) or nuclease treatment (to degrade nucleic acids) [24] (Fig. 3A), suggesting the involvement of heat- and nuclease-resistant metabolites.

We therefore performed metabolomic screening on tissue samples from a breast cancer mouse model with skin involvement. PGE_2_, a known immunosuppressive metabolite that impaired DC function [33, 34], was significantly upregulated in lesional skin (Fig. 3B–D). Consistent with this, enzyme-linked immunosorbent assay (ELISA) and immunofluorescence (IF) analyses confirmed elevated PGE_2_ levels in lesional skin from breast cancer patients compared to non-lesional skin (Fig. 3E, F). Similarly, high concentrations of PGE_2_ were detected in tumor CM, and its concentration was unaffected by heat or nuclease treatment (Fig. 3G). Furthermore, direct exposure of cutaneous DCs on PGE_2_ significantly inhibited the expression of HLA-DR, CD80, and CD86, and impaired both antigen processing and T lymphocyte priming ability (Fig. 3H–K).

Taking together, these data indicate that high levels of PGE_2_ in lesional skin microenvironment contribute to impaired DC activation and function.

### PGE_2_ expression negatively correlated with the infiltration of cutaneous DCs and patient outcomes

**Fig. 4:**
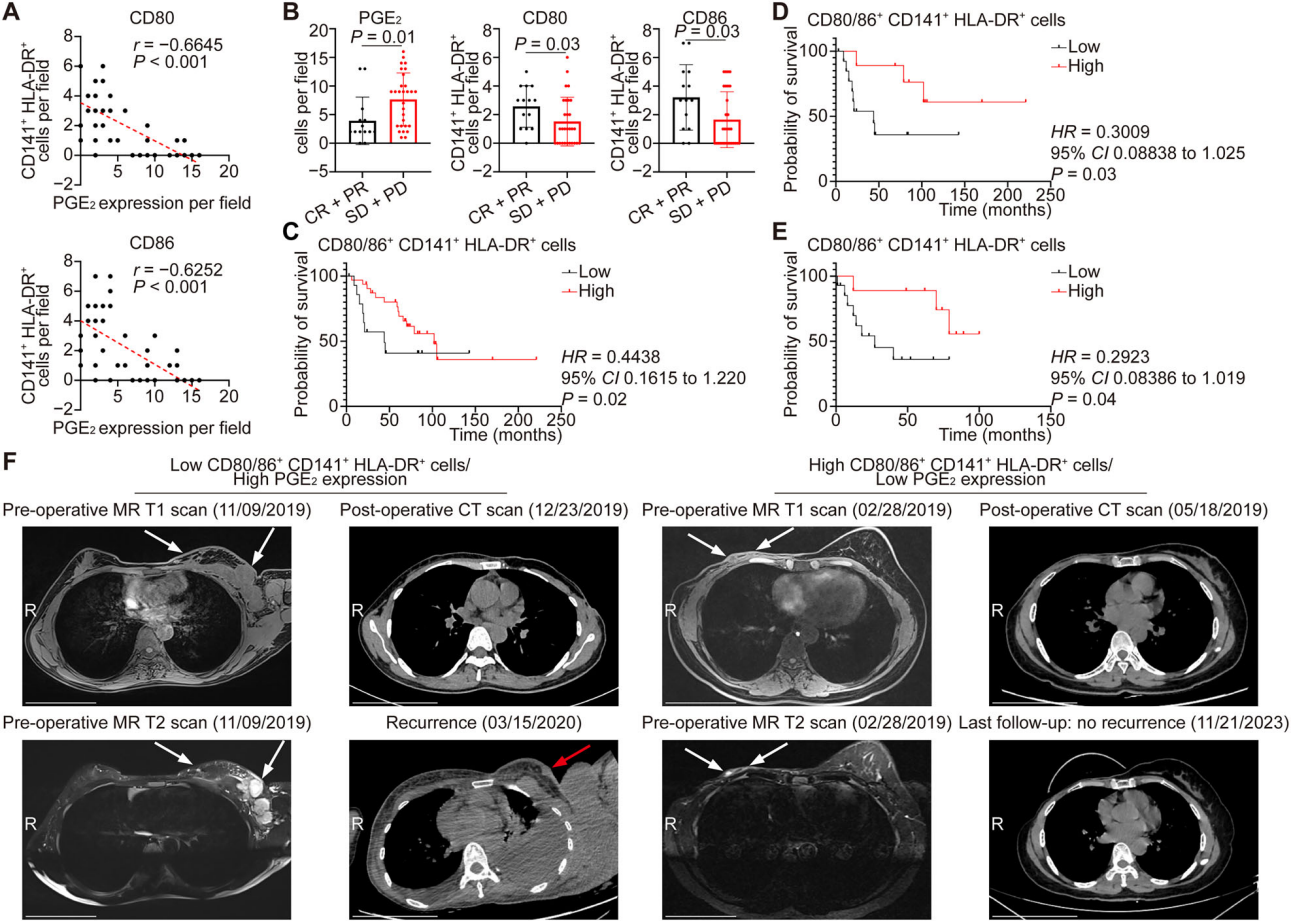
PGE_2_ expression negatively correlated with the infiltration of activated DCs and patient outcomes. **A** The scatter plots showed the correlation between the expression of PGE_2_ and the number of activated cutaneous CD141^+^ DCs. *n* = 47; each dot represented one sample. A linear regression-fitting curve was shown as a red dashed line. **B** The number of PGE_2_^+^ cells or the number of activated cutaneous CD141^+^ DCs with the best treatment response. *n* = 14 and 29 in the CR + PR and SD + PD group, respectively. Four patients were excluded due to missing treatment response data. **C** The Kaplan-Meier plot showed overall survival (OS) of breast cancer patients with or without skin involvement with the high and low number of activated cutaneous CD141^+^ DCs. *n* = 15 and 32 in the low and high group, respectively. The Kaplan-Meier plots showed OS (**D**) and relapse-free survival (RFS) (**E**) in breast cancer patients with skin involvement stratified by high versus low levels of activated cutaneous CD141^+^ DCs. *n* = 14 and 9 in the low and high group, respectively. **F** Representative axial breast and chest magnetic resonance (MR) and computed tomographic (CT) scan images from two breast cancer patients with skin involvement, obtained before and after surgery and during follow-up. One patient (left panel) with less activated DCs and high PGE_2_ expression in skin tissues exhibited a recurrent nodule within 3 months after surgery, while the other patient (right panel) with more activated DCs and low PGE_2_ expression in the skin lesions had no sign of recurrence after a 4.5-year follow-up. White arrows in MR T1- or T2-weighted scan images indicated the malignant nodules before surgery, while the red arrow in the CT scan image indicated a recurrent nodule. R marked the right direction of each image. Scale bar, 10 cm. Data were mean ± SD (**B**); significance was determined using Spearman’s correlation coefficient (*r*) and subsequent *t*-test (**A**), a two-tailed Mann-Whitney’s *U*-test (**B**) or Gehan-Breslow-Wilcoxon test (**C**–**E**). CD cluster of differentiation, CI confidence of interval, CR complete response, HLA human leukocyte antigen, HR hazard ratio, PD progressive disease, PGE_2_ prostaglandin E_2_, PR partial response, SD stable disease.

Clinically, the expression of PGE_2_ exhibited a negative correlation with the infiltration of activated CD141^+^ DCs in the skin tissues of breast cancer patients with or without skin involvement (Spearman’s *r* [95% *CI*], −0.6645 [−0.8023 to −0.4594] and −0.6252 [−0.7770 to −0.4047], respectively; both *P* < 0.001 in CD80^+^ and CD86^+^) (Fig. 4A). Additionally, those patients who exhibited more favorable treatment response had low PGE_2_ expression but high activated CD141^+^ DCs in the skin tissues (*P* = 0.01, 0.03 and 0.03 in PGE_2_, CD80^+^ and CD86^+^, respectively) (Fig. 4B), and importantly, high activated CD141^+^ DCs were associated with improved OS (*P* = 0.02) (Fig. 4C). Among patients with skin involvement, the presence of abundant activated CD141^+^ DCs in skin lesions correlated with both prolonged OS and RFS (*P* = 0.03 and 0.04, respectively) (Fig. 4D, E). Particularly, one breast cancer patient with low activated CD141^+^ DCs and high PGE_2_ expression in the lesional skin experienced a local recurrence within 3 months after the surgery; conversely, the other patient who had abundant activated DCs and low PGE_2_ expression in the skin lesions achieved a 4.5-year RFS after the surgery (Figs. 4F and S4).

### PGE_2_ inhibition relieved skin involvement and restored DC activation in the mouse model

**Fig. 5:**
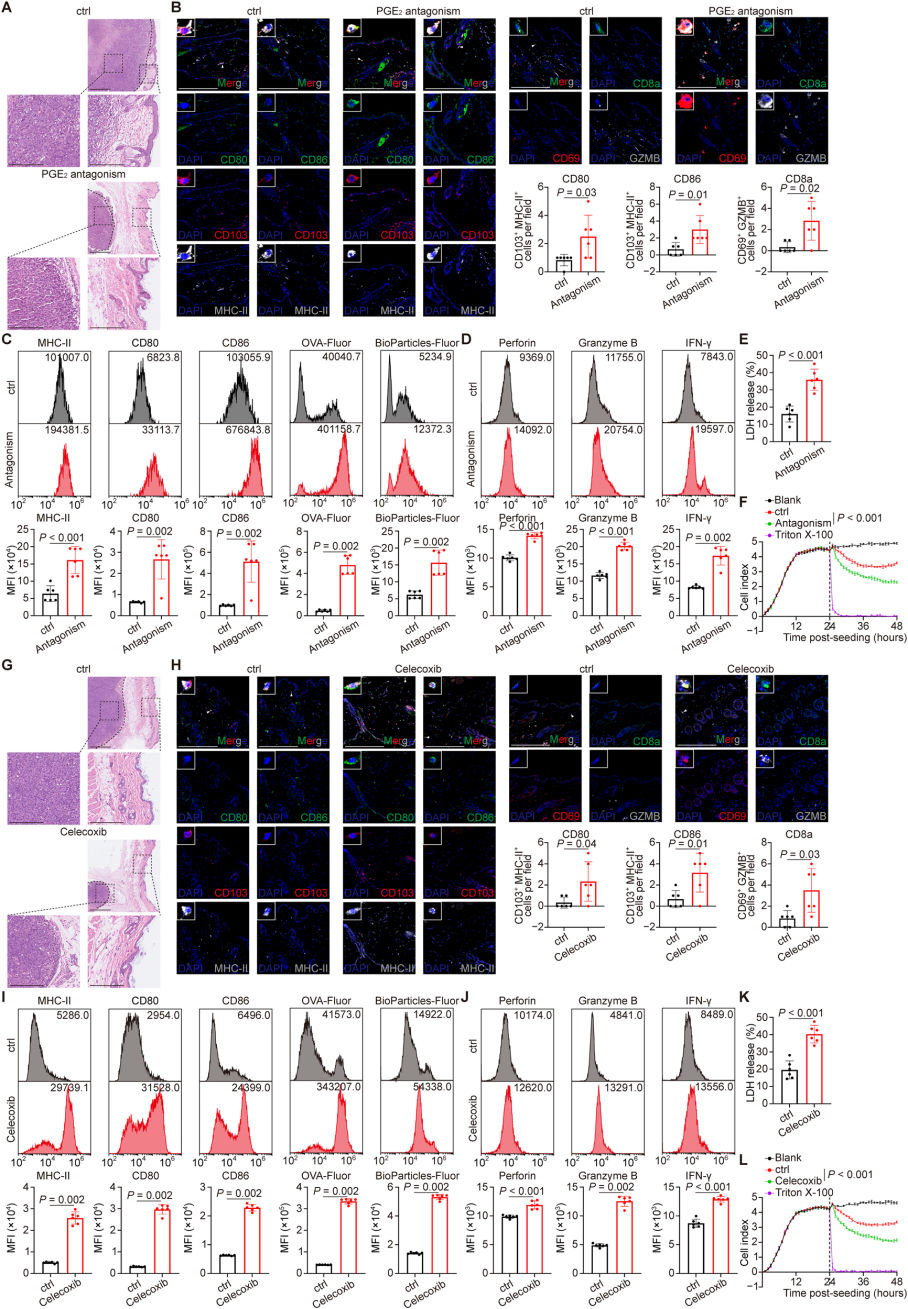
PGE_2_ inhibition relieved skin involvement and restored DC activation and T lymphocyte function in the mouse model. **A**–**F** The breast cancer mouse model with skin involvement was treated with either the control or PGE_2_ antagonism. *n* = 6 mice per group (**A**–**E**) or 3 biological replicates per group (**F**). **A** Representative H&E images depicting skin involvement in the breast cancer mouse model. Black dashed lines showed approximate tumor margins; black dashed squares showed the magnified regions. Scale bar, 500 μm or 250 μm (amplification). **B** Representative immunofluorescent staining of activated DCs and functional T lymphocytes in skin tissues from the indicated breast cancer mouse model. White triangles indicated the cells shown in the upper-left insets in each image. Scale bar, 200 μm. **C** Cutaneous DCs isolated from mice bearing breast cancer that received either the control or PGE_2_ antagonism treatment were subjected to in vitro function assays and analyzed by flow cytometry. Representative flow cytometry plots showed the expression of MHC-Ⅱ, CD80 and CD86, as well as the uptake and processing of fluorescence-conjugated OVA and bioparticles by DCs. **D**–**F** T lymphocytes infiltrating the skin lesions of breast cancer-bearing mice treated with either the control or PGE_2_ antagonism treatment were analyzed by flow cytometry or spectrophotometry. **D** Representative flow cytometry plots showed cytotoxic cytokines expression of T lymphocytes. The cytotoxic effect of T lymphocytes on tumor cells was assessed using LDH release assay (**E**) and real-time impedance monitoring assay (**F**). The black dashed line indicated the time point at which T lymphocytes were added. **G**–**L** The breast cancer mouse model with skin involvement was treated with either the control or celecoxib. *n* = 6 mice per group (**G**–**K**) or 3 biological replicates per group (**L**). **G** Representative H&E images depicting skin involvement in the breast cancer mouse model. Black dashed lines showed approximate tumor margins; black dashed squares showed the magnified regions. Scale bar, 500 μm or 250 μm (amplification). **H** Representative immunofluorescent staining of activated DCs and functional T lymphocytes in skin tissues from the indicated breast cancer mouse model. White triangles indicated the cells shown in the upper-left insets in each image. Scale bar, 200 μm. **I** Cutaneous DCs isolated from mice bearing breast cancer that received either the control or celecoxib were subjected to in vitro function assays and analyzed by flow cytometry. Representative flow cytometry plots showed the expression of MHC-Ⅱ, CD80 and CD86, as well as the uptake and processing of fluorescence-conjugated OVA and bioparticles by DCs. **J**–**L** T lymphocytes infiltrating the skin lesions of breast cancer-bearing mice treated with either the control or celecoxib were analyzed by flow cytometry or spectrophotometry. **J** Representative flow cytometry plots showed cytotoxic cytokines expression of T lymphocytes. The cytotoxic effect of T lymphocytes on tumor cells was assessed using LDH release assay (**K**) and real-time impedance monitoring assay (**L**). The black dashed line indicated the time point at which T lymphocytes were added. Data were mean ± SD; significance was determined using a two-tailed Student’s *t*-test (MHC-Ⅱ in **C**; Perforin and Granzyme B in **D**; **E**; CD86 in **H**; Perforin and IFN-γ in **J**; **K**) or Mann-Whitney’s *U*-test (others in **B**–**D**, **H**–**J**), or one-way analysis of variance (ANOVA) and subsequent Dunnett’s multiple comparisons test at the last time point (**F**, **L**). CD cluster of differentiation, ctrl control, DAPI 4’,6-diamidino-2-phenylindole, Fluor fluorescence, IFN interferon, LDH lactate dehydrogenase, MFI mean fluorescence intensity, MHC major histocompatibility complex, OVA ovalbumin, PGE_2_ prostaglandin E_2_.

To further investigate the impact of PGE_2_ on DC function in vivo, we administered PGE_2_ antagonists (PF-04418948 and L-161,982) to breast cancer-bearing mice with skin involvement [22, 23, 33]. Treatment with these antagonists significantly reduced skin involvement (Fig. 5A) and promoted the infiltration of activated CD103^+^ DCs (the counterparts of human CD141^+^ DCs) and effector CD8^+^ T lymphocytes (expressing CD69 and granzyme B) into lesional skin (Fig. 5B). Flow cytometry confirmed that DCs from PGE_2_ antagonist-treated mice exhibited enhanced activation, antigen processing, and T lymphocyte priming capacity (Figs. 5C and S5A, B), which was accompanied by increased cytotoxicity of tumor-infiltrating CD8^+^ T lymphocytes (Fig. 5D–F). Consistently, the administration of celecoxib, a specific COX-2 inhibitor that blocks PGE_2_ synthesis [24], reduced skin lesions (Fig. 5G), increased the recruitment of activated CD103^+^ DCs and effector CD8^+^ T lymphocytes (Fig. 5H), and augmented the functional capacity of cell types (Figs. 5I–L and S5C, D). The anti-tumor effects of PGE_2_ blockade were not attributed to direct effects on cancer cell proliferation, as neither PGE_2_, PGE_2_ antagonists, nor celecoxib affected tumor cell growth in vitro (Fig. S6). Furthermore, the efficacy of PGE_2_ inhibition was confirmed in a melanoma mouse model, which also showed reduced skin involvement and enhanced DC and T lymphocyte activation (Fig. S7A–F).

These findings demonstrate that PGE_2_ inhibition restricts skin involvement in multiple cancer types by restoring DC and T lymphocyte-mediated anti-tumor immunity.

### Abundant PGE_2_ expression and fewer activated DCs in patients with skin malignancies

**Fig. 6:**
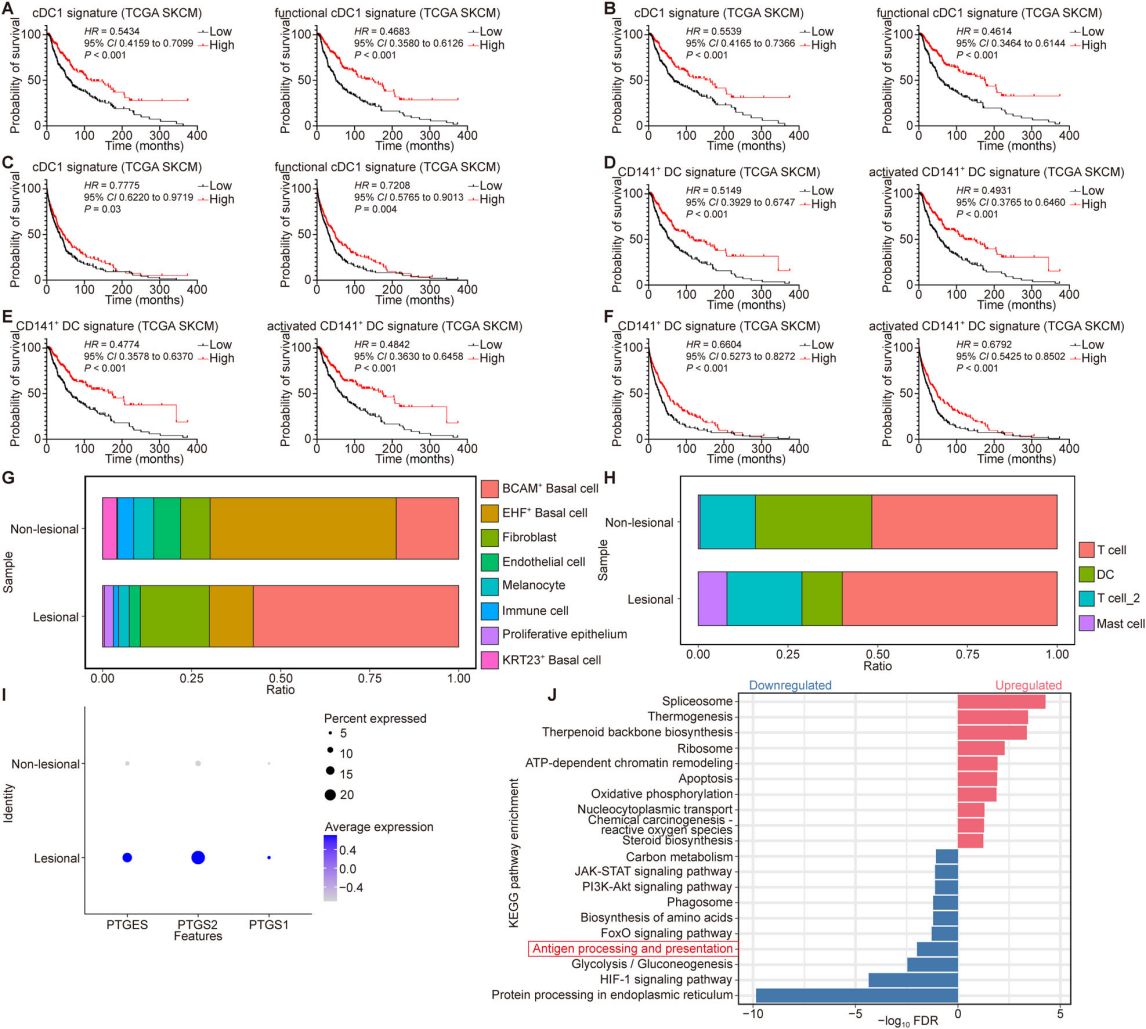
Bioinformatic re-analyses of sequencing datasets of skin cutaneous melanoma and basal cell carcinoma patients. **A**–**F** Re-analyzing the dataset from The Cancer Genome Atlas (TCGA) skin cutaneous melanoma (SKCM). The Kaplan-Meier plots showed OS (**A**), disease-specific survival (DSS) (**B**), and progression-free survival (PFS) (**C**) of patients with higher or lower cDC1 (*n* = 200 and 258, respectively) or functional cDC1 (*n* = 223 and 235, respectively) signature gene expression levels. The Kaplan-Meier plots showed OS (**D**), DSS (**E**), and PFS (**F**) of patients with higher or lower CD141^+^ DC (*n* = 240 and 218, respectively) or activated CD141^+^ DC (*n* = 234 and 224, respectively) signature gene expression levels. **G**–**J** Re-analyzing the publicly available single-cell sequencing dataset of basal cell carcinoma (BCC) patients (GSE141526). The percentage of different clusters of total cells (**G**) and immune cells therein (**H**) in lesional and non-lesional skin of BCC patients. **I** The gene expression of PGE_2_ synthesis-associated enzymes (*PTGES*, *PTGS2* and *PTGS1*) of total cells in lesional and non-lesional skin of BCC patients. **J** KEGG enriched pathways of corresponding cutaneous DCs in lesional and non-lesional skin of BCC patients. Light red colors indicated the upregulated pathways while navy blue colors indicated the downregulated pathways in lesional skin. The red box marked the antigen processing and presentation pathway. Significance was determined using log-rank test (**A**–**F**). CI confidence of interval, DC dendritic cell, FDR false discovery rate, HR hazard ratio, KEGG Kyoto Encyclopedia Genes and Genomes, PGE_2_ prostaglandin E_2_, PTGES prostaglandin E synthase, PTGS prostaglandin-endoperoxide synthase.

Finally, to expand the clinical relevance of PGE_2_-mediated DC dysfunction, we analyzed publicly available datasets of skin malignancies. We derived two established human gene signatures for conventional type 1 DC (cDC1) and functional cDC1 [34, 35], and probed these signatures in tumor gene expression datasets of The Cancer Genome Atlas (TCGA) skin cutaneous melanoma (SKCM) cohort. Intriguingly, we found that a higher expression of either cDC1 signature within skin tumor tissues was significantly correlated with improved OS, DSS and PFS (Fig. 6A–C). Similar results were obtained in survival analyses of gene signatures for CD141^+^ DC and activated CD141^+^ DC (Fig. 6D–F). Notably, both CD141^+^ DC signatures were significant independent predictors of OS, DSS and PFS (Tables S2–S4), highlighting the crucial role of CD141^+^ DCs (cDC1s) in anti-tumor immunity. Moreover, these signatures exhibited negative correlations with the expression of prostaglandin-endoperoxide synthase (*PTGS*, encoding COX) (Fig. S8A, B). Besides, through data mining of the publicly available single-cell sequencing dataset in basal cell carcinoma (BCC) patients (GSE141526) [36], we identified a significant upregulation in the expression of genes encoding for PGE_2_ synthesis in BCC patients, which was accompanied by a decrease in both quantity and antigen processing ability of DCs in lesional skin samples (Figs. 6G–J, S8C–F). Notably, the expression of *PTGS2* (COX-2) showed the most pronounced alterations across all the above analyses, emphasizing the potential application value of its inhibitors like celecoxib. Collectively, these data across distinct skin malignancies substantiated the promising therapeutic potential of targeting PGE_2_ in lesional skin.

## Discussion

Our skin serves as a highly immune-rich tissue that provides defense against exogenous stimuli owing to the complicated networks of the multiplicity of stromal and immune cells, the complexity of hematogenous and lymph vessels as well as the competence of lymph nodes. Dysregulation of skin immunity like DCs contributes to the pathogenesis of various skin inflammable disorders [37]. Considering that the skin is also susceptible to malignancies, such as breast cancer, which often leads to poor patient survival and life quality [8, 38], it becomes imperative to unravel the interaction between DCs in our skin and cancer cells. In this study, we integrated immunostaining, high-throughput sequencing and in vitro experiments to comprehensively characterize the functions of DCs in lesional skin of breast cancer patients. Our findings demonstrated that PGE_2_ suppressed the function of cutaneous DCs, thereby promoting the skin involvement of breast cancer.

Human and mouse DCs have numerous shared characteristics, while some distinctions, like major histocompatibility complex (MHC)-Ⅰ-related molecules CD1a-c, which are exclusively expressed in human DCs and possess the ability to present lipid antigens to T lymphocytes [25], make it challenging to discover clinical findings and interpret data from mice. In our study, isolation and analysis of DCs from the skin tissues of breast cancer patients provide a more precise depiction of their functional status and offer a better insight into the pathogenesis. The limited efficacy of radiotherapy, surgery, and chemotherapy in most breast cancer patients with skin involvement remains an unresolved clinical challenge [8]. Given the pivotal role of DCs in initiating antigen-specific immunity, modulating DCs holds great potential for enhancing cancer immunotherapy [39]. Therapeutic strategies involving delivery of antigens and adjuvants to DCs as well as development of DC vaccines have demonstrated preclinical efficacy in controlling cancer, with several ongoing clinical trials [40–42]. Besides, the COX-2 enzyme inhibitors could effectively decrease the level of PGE_2_. Intriguingly, some preclinical data showed that the COX-2 inhibitor, celecoxib, could boost the efficacy of immunotherapy [24, 43]. And our findings further demonstrate that celecoxib can relieve skin involvement in cancers and restore DC activation. Thus, modulating DCs or the administration of celecoxib may also be applied for treating skin involvement in breast cancer in the future.

A limitation of this study is the restricted cohort size of patients. However, primary DCs from multiple breast cancer patients further merit the value of our study. The further involvement of multiple institutions will provide more persuasive information. Given that celecoxib is widely used in clinical practice, it could be explored in the setting of clinical trials whether targeting PGE_2_ by celecoxib could reverse the immunosuppression of DCs and improve the survival and life quality of breast cancer patients with skin involvement.

## Conclusions

Our study, for the first time, unveils the immunosuppressed status of DCs in lesional skin of breast cancer patients, and functional studies have demonstrated that PGE_2_ mediates the suppression of DCs, highlighting its potential as a therapeutic target for managing breast cancer patients with skin involvement.

## Supplementary information

Supplement

## Data Availability

All data and materials used in the analysis will be made available from the corresponding authors upon reasonable request.
