# Supplementary material for: Prostaglandin E2 induces dendritic cell dysfunction in skin involvement of breast cancer
Source: Cell Death Dis. 2026 Feb 25;17(1):260. doi: 10.1038/s41419-026-08519-1 (PMC12988867; doi:10.1038/s41419-026-08519-1)
Supplement: Supplementary file 1 — Supplement [file 41419_2026_8519_MOESM1_ESM.pdf]

Supplementary files

Title: Prostaglandin E_2 induces dendritic cell dysfunction in skin involvement of breast cancer

Running head: PGE_2 promotes DC dysfunction

Authors:

Jiawen Wang, MD[1,2,3,#], Xiaoming Zhong, MD[1,2,#], Xu Liu, MD[1,2,#], Zhiyun Qian, MM[1,2,#], Jingkun Zhu, MD[1,2], Huayue Lin, PhD[1,2], Jiahui Zhang, MD[1,2], Wei Zhang, MD[1,2], Sicong Du, MD[1,2], Linbin Yang, MD[1,2,*], Man Nie, MD[4,*]

Affiliations:

1 Guangdong Provincial Key Laboratory of Malignant Tumor Epigenetics and Gene Regulation, Guangdong-Hong Kong Joint Laboratory of RNA Medicine, Sun Yat-sen Memorial Hospital, Sun Yat-sen University, Guangzhou, P. R. China.

2 Breast Tumor Center, Sun Yat-sen Memorial Hospital, Sun Yat-sen University, Guangzhou, P. R. China.

3 Department of Thoracic Surgery, Sun Yat-sen Memorial Hospital, Sun Yat-sen University, Guangzhou, P. R. China.

4 Department of Medical Oncology, State Key Laboratory of Oncology in South China, Guangdong Provincial Clinical Research Center for Cancer, Collaborative Innovation Center for Cancer Medicine, Sun Yat-sen University Cancer Center, Guangzhou, P. R. China.

# Drs J. Wang, X. Zhong, X. Liu and Z. Qian contributed equally to this article.

* Drs L. Yang and M. Nie contributed equally to this article as senior authors.

Corresponding authors: Linbin Yang, MD and Man Nie, MD (lead contact)

E-mail: yanglb8@mail.sysu.edu.cn (L. Yang) and nieman@sysucc.org.cn (M. Nie)

This file includes:

Supplementary methods.

Supplementary tables and legends.

Supplementary figures and legends.

Supplementary references.

Supplementary methods

Cell isolation

Cutaneous DCs were isolated from skin tissues as previously described with slight modifications.(1-3) Briefly, fresh skin tissues were cut into smaller pieces using scalpels after carefully removing subcutaneous fat. Then skin pieces were incubated with 2.5 mg/mL dispase II (Roche Diagnostics), 2 mg/mL collagenase type 4 (for human; or type 3 for mouse; both from Worthington Biochem), 0.1 mg/mL DNase I (Roche Diagnostics) and 1 mg/mL hyaluronidase (for mouse only; Sigma-Aldrich) at 37 °C for 2 – 4 hr with frequent shaking. The dissociated cells were obtained by pipetting with serological pipettes and filtering through 70-μm cell strainers.

Primary T lymphocytes were isolated from peripheral blood mononuclear cells (PBMCs, human) or spleen (mouse) using differential density gradient centrifugation as previously described.(4, 5)

Primary breast tumor cells were isolated as previously described with slight modifications.(6) In brief, fresh tumor tissues were cut into smaller pieces using scalpels after carefully removing necroptotic tissues. Then tumor pieces were incubated with 1 mg/mL collagenase type 1, 1 mg/mL collagenase type 3 (both from Worthington Biochem), 1.5 mg/mL hyaluronidase (Sigma-Aldrich) and 0.1 mg/mL DNase I (Roche Diagnostics) at 37 °C for 2 – 3 hr with frequent shaking. The dissociated cells were obtained by pipetting with serological pipettes and filtering through 70-μm cell strainers.

Cell culture

For *in vitro* culture with conditioned medium (CM), half of normal control complete medium was replaced with indicated CM from tumor as previously described.(7) Briefly, heat treatment was achieved by incubating CM at 95 °C for 20 min while nuclease treatment was performed by incubating CM in the presence of 2.5 U/μL benzonase nuclease (Sigma-Aldrich) at 37 °C for 30 min.

Hematoxylin and eosin staining and immunofluorescence

Formalin-fixed paraffin-embedded (FFPE) tissues were sectioned at a thickness of 4 μm. After deparaffinization and rehydration, the slices were stained with hematoxylin and eosin (H&E). For immunofluorescence, heat-induced antigen retrieval was performed before blocking non-specific binding by incubating slices with 5% normal donkey serum (Jackson ImmunoResearch Laboratories) for 1 hr at room temperature. Subsequently, slices were incubated with primary antibodies overnight at 4 °C. Then slices were incubated with Alexa Fluor-conjugated secondary antibodies (Thermo Fisher scientific or Jackson ImmunoResearch Laboratories) for 1 hr at room temperature. 4',6-diamidino-2-phenylindole (DAPI, Sigma-Aldrich) was employed to counterstain nuclei and images were acquired by laser-scanning confocal microscopy (LSM 800, Zeiss or SP8, Leica). At least three different fields per sample were evaluated for quantification and round off mean of them was used for comparison.

The following primary antibodies (clones) were used as manufacturers' instructions (Abcam, otherwise noted): anti-CD3 (SP7), anti-CD20 (SP32), anti-NCAM1 (CD56, EP2567Y), anti-CD66b (G10F5, BioLegend), anti-CD1c (EPR23189-196), anti-Thrombomodulin (CD141, EPR4051), anti-CD14 (SP192), anti-CD80 [EPR1157(2); 16-10A1, Thermo Fisher scientific], anti-CD86 (C86/1146), anti-HLA-DR (TAL 1B5), anti-I-A/I-E (MHC class II, M5/114.15.2, Thermo Fisher scientific), anti-CD103 (EPR22590-27), anti-CD8a (4SM16, Thermo Fisher scientific), anti-CD69 (polyclonal, GeneTex; D-3, Santa Cruz Biotechnology), anti-Granzyme B (polyclonal) and anti-Prostaglandin E_2 (Cayman Chemical).

Flow cytometry

Cells were harvested and washed in protein-free DPBS once. In some cases, fixable viability stain was used to determine the live cells and Human BD Fc Block or anti-mouse CD16/CD32 antibody (Mouse BD Fc Block) prepared in Stain Buffer (all from BD Bioscience) was used to decrease the non-specific binding of antibodies. Primary

antibodies were incubated at 4 °C for 30 min. For intracellular cytokines, cells were further fixed and permeabilized (Thermo Fisher scientific), and primary antibodies were incubated at room temperature for 30 min. Data were acquired by flow cytometer (CytoFLEX S, Beckman Coulter), and analyzed by CytExpert (version 2.5.0.77, Beckman Coulter) or FlowJo (version 10.8.1, Becton Dickinson & Company).(1, 8)

The following primary antibodies (clones) were used as manufacturers' instructions (BD Bioscience, otherwise noted): anti-CD45 (2D1), anti-CD3 (UCHT1), anti-CD8 (RPA-T8), anti-HLA-DR (G46-6), anti-CD14 (M5E2), anti-CD16 (3G8), anti-CD1a (HI149, BioLegend), anti-CD11c (B-ly6), anti-CD26 (BA5b, BioLegend), anti-CD1c (L161, Thermo Fisher scientific), anti-CD141 (1A4), anti-CD80 (L307.4), anti-CD86 [2331 (FUN-1)], anti-Perforin (δG9), anti-Granzyme B (GB11) and anti-IFN-γ (4S.B3); anti-CD45 (30-F11), anti-CD3e (145-2C11), anti-CD8a (53-6.7), anti-EpCAM (CD326, G8.8, BioLegend), anti-CD11c (HL3), anti-I-A/I-E (MHC class II, M5/114.15.2), anti-CD103 (M290), anti-CD11b (M1/70), anti-CD24 (M1/69), anti-CD64 a and b alloantigens (X54-5/7.1), anti-CD80 (16-10A1), anti-CD86 (GL1), anti-Perforin (S16009A, BioLegend), anti-Granzyme B (NGZB, Thermo Fisher scientific) and anti-IFN-γ (XMG1.2).

The gating strategies for human or mouse samples are provided in Supplementary figures as previously described.(9)

Antigen processing

Alexa Fluor-conjugated ovalbumin (OVA)(10) and pHrodo *E. coli* BioParticles(11) (both from Thermo Fisher scientific) were used as manufacturer's instructions with slight modifications. In brief, DCs were harvested, washed with PBS and resuspended in Live Cell Imaging Solution (Thermo Fisher scientific). Fluorescence-conjugated OVA or bioparticles were added at a final working concentration of 20 μg/mL and 100 mg/mL, respectively. After incubation at 37 °C for 1 hr, DCs were harvested and analyzed by flow cytometry.

Mixed leukocyte reaction

Proliferation and cytotoxic cytokines expression of T lymphocytes primed by allogeneic DCs based on mixed leukocyte reaction (MLR) assays were performed, as previously described with slight modifications, to reflect the DC priming function.(1, 12) (13) Briefly, allogeneic T lymphocytes were added to DCs at a ratio of 5/1 (T/DCs) and co-cultured for 5 d. At the end of the culture, suspended T lymphocytes were harvested and analyzed by flow cytometry. For proliferation analyses, aforehand fluorescence labeling to T lymphocytes was achieved by CellTrace CFSE reagent (Thermo Fisher scientific) as manufacturer's instructions. For cytotoxic cytokines expression analyses, T lymphocytes activation was achieved by adding Leukocyte Activation Cocktail and BD Golgi Plug (BD Bioscience) at a final dilution of 1/100, 4 hr before harvest as manufacturer's instructions.

Enzyme-linked immunosorbent assay

PGE_2 concentration was determined by Prostaglandin E_2 Express ELISA Kit (Cayman Chemical) following manufacturer's instructions as previously described.(14) Briefly, tissue samples were harvested and homogenized in PBS supplemented with 1 mM EDTA (Thermo Fisher scientific) and 10 μM indomethacin (Sigma-Aldrich); then PGE_2 content in the supernatant of the lysates or indicated CM was quantified by enzyme-linked immunosorbent assay (ELISA).

Cell proliferation

Cell proliferation was determined by Cell Counting Kit-8 (CCK8, Dojindo Laboratories) following manufacturer's instructions as previously described.(15) Briefly, tumor cells were seeded in 96-well plates and measured by CCK8 daily.

LDH release assay

The activities of lactate dehydrogenase (LDH) were used to measure cell cytotoxicity using a LDH Cytotoxicity Assay Kit (Beyotime Biotechnology) following manufacturer's instructions as previously described with slight modifications.(16) In brief, 1×10^5 per mL tumor cells in 100 μL DMEM medium were seeded into 96-well plate and co-cultured with the $CD8^+$ T lymphocytes primed by DCs or isolated from the experimental mouse model (effector/target ratio, E/T = 10/1) at 37 °C overnight. Afterwards, the supernatant was collected into a new 96-well plate and incubated with reaction mixture for 30 min in the dark. Then, the absorbance was detected at 490 nm and 680 nm after adding stop solution. To acquire maximum release, cells were treated with 10% Triton X-100 (Sigma-Aldrich). The percentage of LDH cytotoxicity was calculated from measured LDH activity using the manufacturer's recommended formula: LDH cytotoxicity (%) = ((experimental value A490) − (spontaneous release A490)) / ((maximum release A490) − (spontaneous release A490)) × 100.

Real-time impedance monitoring assay

xCELLigence RTCA eSight instrument (Agilent Technologies) was used to monitor survival of cells and further measure cell cytotoxicity based on measurements of cell impedance following manufacturer's instructions as previously described.(17, 18) Tumor cells were seeded at a density of 1×10^4 cells/well in preheated medium into an E-plate 96 (Agilent Technologies). Post-seeding, the E-plate was transferred to the monitor system inside a cell incubator and incubated for 24 hr. Data recording was set to 1-hour intervals over 48 hr and changes in impedance were reported as a Cell index (CI). After 24 hr, indicated T lymphocytes were added to the tumor cell cultures (E/T = 10/1). Negative control wells contained tumor cells alone and positive control wells were treated with Triton X-100 (Sigma-Aldrich). Tumor and T lymphocytes were co-cultured for another 24 hr.

Bulk RNA sequencing and data processing

Total RNA was extracted from cutaneous DCs of breast cancer patients with and without skin involvement using TRIzol reagent (Thermo Fisher scientific). Eukaryotic mRNA was enriched by Oligo(dT) beads. Then the enriched mRNA was fragmented into short fragments using fragmentation buffer and reversely transcribed into cDNA using NEBNext Ultra RNA Library Prep Kit for Illumina (New England Biolabs). Illumina Novaseq6000 was applied for the sequence of the resulting cDNA library. To get high quality clean reads, raw sequencing reads were further filtered by fastp (version 0.18.0). High-quality reads were aligned to the reference genome using HISAT (version 2.2.4) with "-rna-strandness RF" and other parameters set as a default.(19) Gene expression datasets were normalized using RNA-seq by expectation-maximization (RSEM).

Data was processed in RStudio (version 2024.04.1) based on R (version 4.4.0). Differential expression analysis was conducted using "DESeq2" package (version 1.44.0). Genes with false discovery rate (FDR) < 0.05 and log2 fold change (FC) > 1 (up) or < -1 (down) were considered as differentially expressed genes (DEGs) for further Kyoto Encyclopedia Genes and Genomes (KEGG) enrichment analysis.

Single-cell RNA-seq data processing

Single-cell RNA-seq data was downloaded from gene expression omnibus (GEO, https://www.ncbi.nlm.nih.gov/geo/) under the accession number GSE141526.(20, 21) All analyses were conducted using R (version 4.4.0) and "Seurat" package (version 5.1.0).(22) To remove poor-quality cells and doublets, cells were excluded according to the number of genes, unique molecular identifiers (UMIs) and the percentage of mitochondrial genes. Consequently, 56077 single cells [38374 from 4 lesional skin samples of basal cell carcinoma (BCC) and 17703 from 2 non-lesional skin samples] were selected for the following analysis. Harmony (version 1.2.0) was employed to aggregate all samples for minimizing the potential batch effect. Then, cells were clustered using FindNeighbors and FindClusters. Uniform Manifold Approximation

and Projection (UMAP) was performed to visualize the clustering results in two dimensions using the RunUMAP function in Seurat. Marker genes for the individual clusters were identified using FindAllMarkers. For marker genes between lesional and non-lesional skin in DCs, FindMarkers function was applied and genes with $P < 0.05$ and log2 FC > 0.5 (up) or < −0.5 (down) were chosen for further KEGG enrichment analysis.

Metabolomics and data processing

Sample preparation

The tissue samples from breast cancer with or without skin involvement were immediately snap-frozen in liquid nitrogen immediately after surgical resection. Then approximately 80 mg of each tissue sample was dissected on dry ice and transferred into a 2-mL Eppendorf tube. The tissue samples with 200 μL of H_2O and five ceramic beads were homogenized using the homogenizer. 800 μL methanol/acetonitrile (1:1, v/v) were added to homogenized solution for metabolite extraction. The mixture was centrifuged at 14,000 g and 4 °C for 15 min. The supernatant was dried in a vacuum centrifuge. For LC-MS analysis, the samples were re-dissolved in 100 μL acetonitrile/water (1:1, v/v) solvent.

LC-MS analysis

Analysis was performed using an UHPLC (1290 Infinity LC, Agilent Technologies) coupled to a quadrupole time-of-flight (AB Sciex TripleTOF 6600) in Shanghai Applied Protein Technology Co., Ltd. For HILIC separation, samples were analyzed using a 2.1 mm × 100 mm ACQUIY UPLC BEH 1.7 μm column (Waters). In both ESI positive and negative modes, the mobile phase contained A = 25 mM ammonium acetate and 25 mM ammonium hydroxide in water and B = acetonitrile. The gradient was 85% B for 1 min and was linearly reduced to 65% in 11 min, and then was reduced to 40% in 0.1 min and kept for 4 min, and then increased to 85% in 0.1 min, with a 5 min re-equilibration period employed. For RPLC separation, a 2.1 mm × 100 mm

ACQUIY UPLC HSS T3 1.8 μm column (Waters) was used. In ESI positive mode, the mobile phase contained A = water with 0.1% formic acid and B = acetonitrile with 0.1% formic acid; and in ESI negative mode, the mobile phase contained A = 0.5 mM ammonium fluoride in water and B = acetonitrile. The gradient was 1% B for 1.5 min and was linearly increased to 99% in 11.5 min and kept for 3.5 min. Then it was reduced to 1% in 0.1 min and a 3.4 min of re-equilibration period was employed. The gradients were at a flow rate of 0.3 mL/min, and the column temperatures were kept constant at 25 °C. A 2 μL aliquot of each sample was injected. The ESI source conditions were set as follows: Ion Source Gas1 (Gas1) as 60, Ion Source Gas2 (Gas2) as 60, curtain gas (CUR) as 30, source temperature: 600 °C, IonSpray Voltage Floating (ISVF) ± 5,500 V. In MS only acquisition, the instrument was set to acquire over the m/z range 60 – 1000 Da, and the accumulation time for TOF MS scan was set at 0.20 s/spectra. In auto MS/MS acquisition, the instrument was set to acquire over the m/z range 25 – 1000 Da, and the accumulation time for product ion scan was set at 0.05 s/spectra. The product ion scan is acquired using information dependent acquisition (IDA) with high sensitivity mode selected. The parameters were set as follows: the collision energy (CE) was fixed at 35 V with ± 15 eV; declustering potential (DP), 60 V (+) and −60 V (−); exclude isotopes within 4 Da, candidate ions to monitor per cycle: 10.

Data processing

The raw MS data were converted to MzXML files using ProteoWizard MSConvert before importing into freely available XCMS software. For peak picking, the following parameters were used: centWave m/z = 10 ppm, peakwidth = c (10, 60), prefilter = c (10, 100). For peak grouping, bw = 5, mzwid = 0.025, minfrac = 0.5 were used. CAMERA (Collection of Algorithms of MEtabolite pRofile Annotation) was used for annotation of isotopes and adducts. In the extracted ion features, only the variables having more than 50% of the nonzero measurement values in at least one group were kept. Compound identification of metabolites was performed by comparing of accuracy m/z value (< 10 ppm), and MS/MS spectra with an in-house database established with

available authentic standards. The missing data were filled by KNN (K-Nearest Neighbor) method, and the extreme values were deleted. Finally, the total peak area of the data was normalized to ensure the parallelism between samples and metabolites. For following analysis, the detected metabolites were uploaded into the statistical analysis [one-factor] package in the online MetaboAnalyst 6.0.(23) In our study, features (40%) were filtered based on interquartile range (IQR). Normalization by sum, log transformation (base 10) and auto scaling were carried out for sample normalization, data transformation and scaling. T-tests were used to calculate *P*-values. Then volcano plot was performed and metabolites with $P < 0.05$ and log2 fold change (FC) > 1 (up) or < −1 (down) were considered as differentially expressed features. To evaluate the separation between two groups, partial least squares discriminant analysis (PLS-DA) was performed. For heatmap visualization, clustering was performed using Ward's method and distance measure was performed using Euclidean's method.

Bioinformatic analysis of cancer patient data

RSEM normalized expression datasets from The Cancer Genome Atlas (TCGA) were downloaded from UCSC Xena (https://xena.ucsc.edu/). For generation of gene expression signatures, normalized expression values were log2-transformed and ranked by the mean expression value of signature genes. The following gene signatures were used: $CD141^+$ DC signature (*HLA-DRA*, *HLA-DRB1*, *HLA-DRB5* and *THBD*), activated $CD141^+$ DC signature (*HLA-DRA*, *HLA-DRB1*, *HLA-DRB5*, *THBD*, *CD80* and *CD86*), cDC1 signature (*XCR1*, *CLNK*, *BATF3* and *CLEC9A*), functional cDC1 signature (*XCR1*, *CLNK*, *BATF3*, *CLEC9A*, *IRF8*, *CXCL9*, *CXCL10*, *CXCL11*, *IL12B*, *IL27* and *TNF*), natural killer (NK) cell signature (*NCR1*, *NCR3*, *KLRB1*, *CD160* and *PRF1*), cytotoxic T lymphocyte (CTL) signature (*CD8A*, *CD8B*, *GZMB*, *GZMA* and *PRF1*) and interferon gene signature (IGS; *RSAD2*, *IFI44*, *IFI44L*, *IFI27*, *IFI6*, *MxA*, *OAS1* and *ISG15*).(14, 24-26) Overall survival (OS), disease-specific survival (DSS) and progression-free survival (PFS) analyses were analyzed by stratifying samples into

high and low expression groups based on the mean expression levels of the indicated signature genes, with results presented as Kaplan-Meier curves and significances determined using log-rank tests.(14, 24) Cox proportional hazard regression models were applied using the Enter method for univariable analysis and the Stepwise Forward likelihood ratio (LR) method for multivariable analysis.

Supplementary tables and legends

Table S1 Characteristics of breast cancer patients

Variables	non-SI (*n* = 24)	SI (*n* = 23)	*P*-value
Sex, No. (%)			> 0.99
Female	24 (100)	23 (100)	
Age at diagnosis, median (range), yr	51.5 (24 – 77)	47 (25 – 77)	0.63
ER status, No. (%)			0.19
Negative	4 (17)	8 (35)	
Positive	20 (83)	15 (65)	
PgR status, No. (%)			0.77
Negative	12 (50)	13 (57)	
Positive	12 (50)	10 (43)	
HER2 status, No. (%)			> 0.99
Negative	16 (67)	16 (70)	
Positive	8 (33)	7 (30)	
Ki67 rate, No. (%)			0.58
≤ 14%	2 (8)	2 (9)	
> 14%	22 (92)	20 (87)	
Unknown	0	1 (4)	
Histology, No. (%)			0.33
Ductal	22 (92)	19 (82)	
Papillary	0	2 (9)	
Others	2 (8)	2 (9)	
Histologic grade, No. (%)			0.46
II	5 (21)	5 (22)	
III	16 (67)	12 (52)	
Unknown	3 (12)	6 (26)	
Surgery, No. (%)			0.14
Breast-conserving surgery	10 (42)	5 (22)	
Modified radical mastectomy	14 (58)	18 (78)	
Chemotherapy, No. (%)	20 (83)	21 (91)	0.67
HER2-targeted therapy, No. (%)	6 (25)	7 (30)	0.75
Endocrine therapy, No. (%)	17 (71)	14 (61)	0.55
Radiation therapy, No. (%)	14 (58)	13 (57)	> 0.99
Clinical manifestation of skin lesions, No. (%)			NA
Nodules	NA	21 (91)	
Carcinoma erysipelatoides	NA	2 (9)	

Table S1 Characteristics of breast cancer patients.

The characteristics of breast cancer patients with or without skin involvement.

≥ 1% was employed as the cut-off for ER and PgR expression. Significance was determined using a two-tailed Mann-Whitney's *U*-test (for age at diagnosis), chi-square test or Fisher's precision probability test (for categorical variables).

Abbreviations: ER, estrogen receptor; HER2, human epidermal growth factor receptor 2; NA, not applicable; non-SI, breast cancer patients without skin involvement; PgR, progesterone receptor; SI, breast cancer patients with skin involvement; yr, year(s).

Table S2 Cox proportional hazard regression analysis of overall survival (OS) in the dataset from The Cancer Genome Atlas (TCGA) skin cutaneous melanoma (SKCM)

(A) Univariable Cox proportional hazard regression analysis

**Variables**	**HR (95% *CI*)**	***P*-value**
Sex (female vs. male)	1.176 (0.882 – 1.568)	0.270
Age at diagnosis, yr (< 58 vs. ≥ 58)	1.603 (1.216 – 2.114)	**0.001**
pT (0 – T 2 vs. T 3 – 4)	2.018 (1.534 – 2.655)	**< 0.001**
pN (0 vs. N 1 – 3)	1.708 (1.288 – 2.265)	**< 0.001**
Breslow thickness, mm (≤ 1 vs. > 1)	1.624 (1.213 – 2.174)	**0.001**
CD141$^+$ DC (low vs. high)	0.513 (0.390 – 0.674)	**< 0.001**
activated CD141$^+$ DC (low vs. high)	0.490 (0.373 – 0.645)	**< 0.001**
CTL (low vs. high)	0.525 (0.399 – 0.692)	**< 0.001**
NK (low vs. high)	0543 (0.412 – 0.715)	**< 0.001**
IGS (low vs. high)	0.551 (0.419 – 0.723)	**< 0.001**

(B) Multivariable Cox proportional hazard regression analysis regarding CD141$^+$ DC signature

**Variables**	**HR (95% *CI*)**	***P*-value**
Age at diagnosis, yr (< 58 vs. ≥ 58)	1.377 (1.031 – 1.840)	**0.030**
pT (0 – T 2 vs. T 3 – 4)	1.717 (1.290 – 2.285)	**< 0.001**
pN (0 vs. N 1 – 3)	1.802 (1.355 – 2.395)	**< 0.001**
Breslow thickness, mm (≤ 1 vs. > 1)		0.823
CD141$^+$ DC (low vs. high)	0.586 (0.436 – 0.787)	**< 0.001**
CTL (low vs. high)		0.319
NK (low vs. high)		0.492
IGS (low vs. high)	0.720 (0.536 – 0.968)	**0.030**

(C) Multivariable Cox proportional hazard regression analysis regarding activated CD141^{+} DC signature

**Variables**	**HR (95% *CI*)**	***P*-value**
Age at diagnosis, yr (< 58 vs. ≥ 58)	1.383 (1.035 – 1.848)	**0.028**
pT (0 – T 2 vs. T 3 – 4)	1.680 (1.261 – 2.238)	**< 0.001**
pN (0 vs. N 1 – 3)	1.820 (1.369 – 2.420)	**< 0.001**
Breslow thickness, mm (≤ 1 vs. > 1)		0.873
activated CD141^{+} DC (low vs. high)	0.561 (0.416 – 0.757)	**< 0.001**
CTL (low vs. high)		0.630
NK (low vs. high)		0.832
IGS (low vs. high)	0.732 (0.545 – 0.985)	**0.039**

Table S2 Cox proportional hazard regression analysis of overall survival (OS) in the dataset from The Cancer Genome Atlas (TCGA) skin cutaneous melanoma (SKCM).

(A-C) The univariable and multivariable Cox proportional hazard regression analysis of OS in the dataset from TCGA SKCM. **(A)** Univariable Cox proportional hazard regression analysis. **(B)** Multivariable Cox proportional hazard regression analysis regarding $CD141^+$ DC signature. **(C)** Multivariable Cox proportional hazard regression analysis regarding activated $CD141^+$ DC signature.

Significance was determined using Cox proportional hazard regression model with the Enter method (for univariable analysis) or Stepwise Forward likelihood ratio (LR) method (for multivariable analysis).

The *P*-values in bold represent < 0.05 and corresponding HRs (95% *CI*s) were reported.

Abbreviations: CI, confidence of interval; CTL, cytotoxic T lymphocyte; DC, dendritic cell; HR, hazard ratio; NK, natural killer; IGS, interferon gene signature; pN, pathological N (node) stage; pT, pathological T (tumor) stage; yr, year(s).

Table S3 Cox proportional hazard regression analysis of disease-specific survival (DSS) in the dataset from TCGA SKCM

(A) Univariable Cox proportional hazard regression analysis

Variables	HR (95% *CI*)	*P*-value
Sex (female vs. male)	1.142 (0.842 – 1.551)	0.393
Age at diagnosis, yr (< 58 vs. ≥ 58)	1.524 (1.135 – 2.048)	**0.005**
pT (0 – T 2 vs. T 3 – 4)	1.930 (1.442 – 2.584)	**< 0.001**
pN (0 vs. N 1 – 3)	1.670 (1.235 – 2.260)	**0.001**
Breslow thickness, mm (≤ 1 vs. > 1)	1.629 (1.193 – 2.223)	**0.002**
$CD141^+$ DC (low vs. high)	0.475 (0.355 – 0.637)	**< 0.001**
activated $CD141^+$ DC (low vs. high)	0.481 (0.359 – 0.645)	**< 0.001**
CTL (low vs. high)	0.514 (0.383 – 0.689)	**< 0.001**
NK (low vs. high)	0527 (0.393 – 0.708)	**< 0.001**
IGS (low vs. high)	0.569 (0.426 – 0.761)	**< 0.001**

(B) Multivariable Cox proportional hazard regression analysis regarding $CD141^+$ DC signature

Variables	HR (95% *CI*)	*P*-value
Age at diagnosis, yr (< 58 vs. ≥ 58)	1.372 (1.006 – 1.870)	**0.046**
pT (0 – T 2 vs. T 3 – 4)	1.657 (1.220 – 2.251)	**0.001**
pN (0 vs. N 1 – 3)	1.751 (1.291 – 2.375)	**< 0.001**
Breslow thickness, mm (≤ 1 vs. > 1)		0.647
$CD141^+$ DC (low vs. high)	0.477 (0.356 – 0.641)	**< 0.001**
CTL (low vs. high)		0.257
NK (low vs. high)		0.434
IGS (low vs. high)		0.104

(C) Multivariable Cox proportional hazard regression analysis regarding activated CD141$^+$ DC signature

Variables	HR (95% *CI*)	*P*-value
Age at diagnosis, yr (< 58 vs. ≥ 58)	1.370 (1.005 – 1.866)	**0.046**
pT (0 – T 2 vs. T 3 – 4)	1.617 (1.189 – 2.198)	**0.002**
pN (0 vs. N 1 – 3)	1.771 (1.306 – 2.403)	**< 0.001**
Breslow thickness, mm (≤ 1 vs. > 1)		0.681
activated CD141$^+$ DC (low vs. high)	0.486 (0.362 – 0.654)	**< 0.001**
CTL (low vs. high)		0.396
NK (low vs. high)		0.602
IGS (low vs. high)		0.095

Table S3 Cox proportional hazard regression analysis of disease-specific survival (DSS) in the dataset from TCGA SKCM.

(A-C) The univariable and multivariable Cox proportional hazard regression analysis of DSS in the dataset from TCGA SKCM. **(A)** Univariable Cox proportional hazard regression analysis. **(B)** Multivariable Cox proportional hazard regression analysis regarding $CD141^+$ DC signature. **(C)** Multivariable Cox proportional hazard regression analysis regarding activated $CD141^+$ DC signature.

Significance was determined using Cox proportional hazard regression model with the Enter method (for univariable analysis) or Stepwise Forward likelihood ratio (LR) method (for multivariable analysis).

The *P*-values in bold represent < 0.05 and corresponding HRs (95% *CI*s) were reported.

Abbreviations: CI, confidence of interval; CTL, cytotoxic T lymphocyte; DC, dendritic cell; HR, hazard ratio; NK, natural killer; IGS, interferon gene signature; pN, pathological N (node) stage; pT, pathological T (tumor) stage; yr, year(s).

Table S4 Cox proportional hazard regression analysis of progression-free survival (PFS) in the dataset from TCGA SKCM

(A) Univariable Cox proportional hazard regression analysis

Variables	HR (95% *CI*)	*P*-value
Sex (female vs. male)	1.038 (0.821 – 1.311)	0.757
Age at diagnosis, yr (< 58 vs. ≥ 58)	1.729 (1.360 – 2.196)	**< 0.001**
pT (0 – T 2 vs. T 3 – 4)	1.585 (1.255 – 2.001)	**< 0.001**
pN (0 vs. N 1 – 3)	1.861 (1.474 – 2.350)	**< 0.001**
Breslow thickness, mm (≤ 1 vs. > 1)	1.305 (1.028 – 1.658)	**0.029**
CD141^{+} DC (low vs. high)	0.654 (0.522 – 0.820)	**< 0.001**
activated CD141^{+} DC (low vs. high)	0.672 (0.536 – 0.843)	**0.001**
CTL (low vs. high)	0.738 (0.589 – 0.924)	**0.008**
NK (low vs. high)	0.733 (0.585 – 0.919)	**0.007**
IGS (low vs. high)	0.795 (0.636 – 0.995)	**0.045**

(B) Multivariable Cox proportional hazard regression analysis regarding CD141^{+} DC signature

Variables	HR (95% *CI*)	*P*-value
Age at diagnosis, yr (< 58 vs. ≥ 58)	1.724 (1.341 – 2.217)	**< 0.001**
pT (0 – T 2 vs. T 3 – 4)	1.311 (1.028 – 1.672)	**0.029**
pN (0 vs. N 1 – 3)	1.961 (1.550 – 2.481)	**< 0.001**
Breslow thickness, mm (≤ 1 vs. > 1)		0.680
CD141^{+} DC (low vs. high)	0.657 (0.523 – 0.825)	**< 0.001**
CTL (low vs. high)		0.953
NK (low vs. high)		0.913
IGS (low vs. high)		0.740

(C) Multivariable Cox proportional hazard regression analysis regarding activated CD141$^+$ DC signature

**Variables**	**HR (95% *CI*)**	***P*-value**
Age at diagnosis, yr (< 58 vs. ≥ 58)	1.731 (1.347 – 2.226)	**< 0.001**
pT (0 – T 2 vs. T 3 – 4)	1.282 (1.003 – 1.639)	**0.047**
pN (0 vs. N 1 – 3)	1.983 (1.567 – 2.511)	**< 0.001**
Breslow thickness, mm (≤ 1 vs. > 1)		0.647
activated CD141$^+$ DC (low vs. high)	0.675 (0.537 – 0.849)	**0.001**
CTL (low vs. high)		0.831
NK (low vs. high)		0.806
IGS (low vs. high)		0.802

Table S4 Cox proportional hazard regression analysis of progression-free survival (PFS) in the dataset from TCGA SKCM.

(A-C) The univariable and multivariable Cox proportional hazard regression analysis of PFI in the dataset from TCGA SKCM. **(A)** Univariable Cox proportional hazard regression analysis. **(B)** Multivariable Cox proportional hazard regression analysis regarding $CD141^+$ DC signature. **(C)** Multivariable Cox proportional hazard regression analysis regarding activated $CD141^+$ DC signature.

Significance was determined using Cox proportional hazard regression model with the Enter method (for univariable analysis) or Stepwise Forward likelihood ratio (LR) method (for multivariable analysis).

The *P*-values in bold represent < 0.05 and corresponding HRs (95% *CI*s) were reported.

Abbreviations: CI, confidence of interval; CTL, cytotoxic T lymphocyte; DC, dendritic cell; HR, hazard ratio; NK, natural killer; IGS, interferon gene signature; pN, pathological N (node) stage; pT, pathological T (tumor) stage; yr, year(s).

Supplementary figures and legends

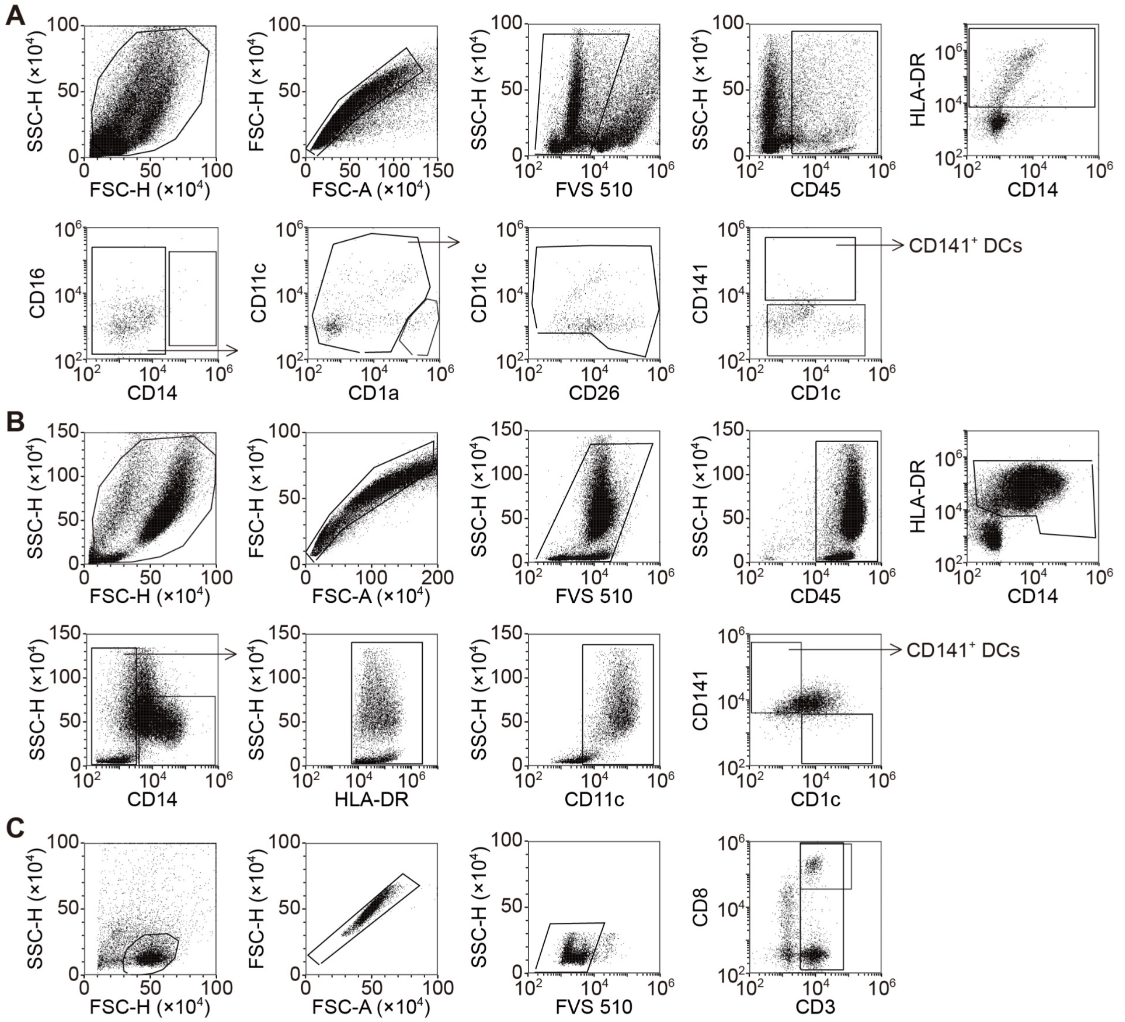

Figure S1 Flow cytometry gating strategies for human samples.

(A, B) The DCs were isolated from the lesional and non-lesional skin tissues of breast cancer patients, applied to *in vitro* function assays, harvested and stained for specific markers in the presence of Human BD Fc Block and analyzed by flow cytometry. **(A)** Representative flow cytometry plots showed a typical gating strategy to identify DCs. Single viable $CD45^+$ immune cells were first identified as follows: forward scatter (FSC) and side scatter (SSC) to identify most cells and exclude debris; FSC height-area ratio to exclude doublets; Fixable viability stain $(FVS)^-$ to identify viable cells; and subsequent $CD45^+$ to identify leukocytes. Single viable $CD45^+$ immune cells were further gated to identify HLA-DR^+ cells and $CD141^+$HLA-DR^+ DCs were then identified as follows: $CD14^-$ to exclude macrophages; $CD1a^-$ to exclude Langerhans

cells (LCs); CD11c and CD26 to identify DCs; CD141 and CD1c to identify CD141$^+$ DCs. **(B)** Representative flow cytometry plots showed a modified gating strategy to identify DCs in the absence of CD1a, CD16 and CD26 because of the limited channels of flow cytometer (especially for CD80 and CD86 expression analyses). Single viable CD45$^+$ immune cells were first identified as (**A**) described and were further gated as follows: CD14 and HLA-DR to identify HLA-DR$^+$ cells; CD14$^-$ to exclude macrophages; HLA-DR and CD11c to identify DCs; CD141 and CD1c to identify CD141$^+$ DCs. **(C)** The DCs were isolated from the lesional and non-lesional skin tissues of breast cancer patients and applied to *in vitro* mixed leukocyte reaction (MLR, T lymphocyte priming) assays; suspended T lymphocytes were harvested, stained for specific markers and analyzed by flow cytometry. Representative flow cytometry plots showed a typical gating strategy to identify T lymphocytes. Single viable cells were first identified as (**A**) described, but no CD45 gating was previously used because of aforehand processes of T lymphocytes isolation. T lymphocytes were further identified as follows: CD3$^+$ to identify total T lymphocytes (for proliferation analyses); CD8$^+$ to identify cytotoxic T lymphocytes among them (for cytotoxic cytokines expression analyses). CD, cluster of differentiation; DC, dendritic cell; FSC, forward scatter; FVS, Fixable viability stain; HLA, human leukocyte antigen; SSC, side scatter.

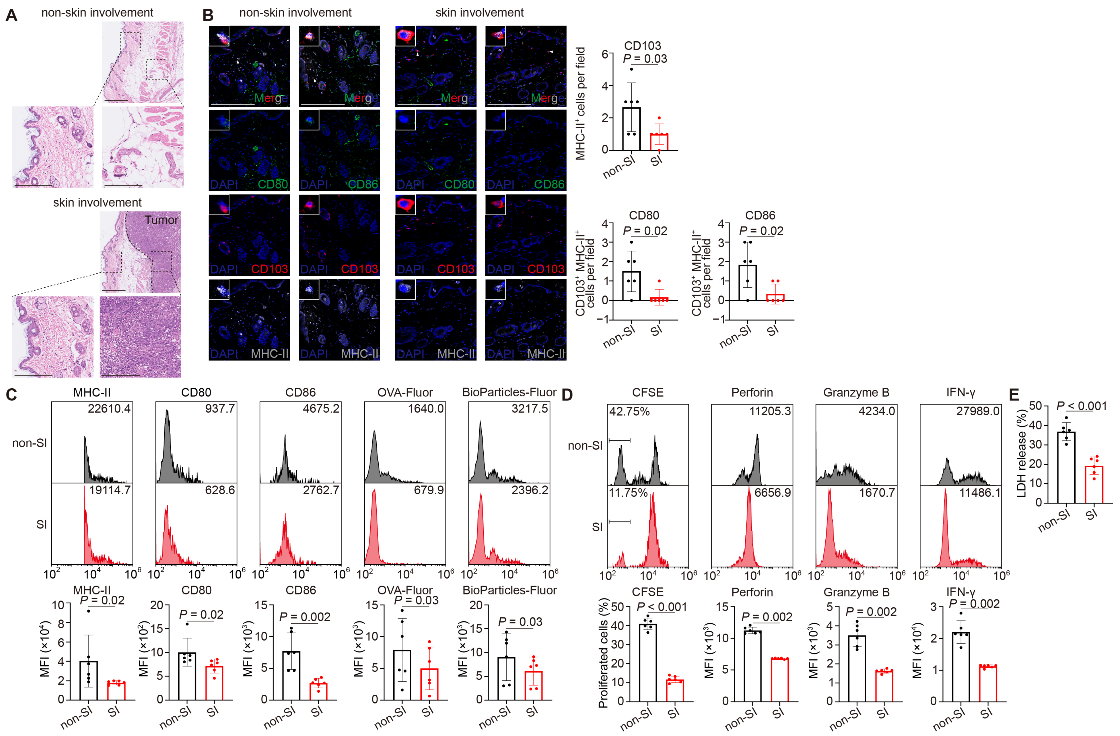

Figure S2 Activation and functions of DCs in the experimental mouse model.

(**A**) Representative H&E images of skin involvement in the experimental mouse model**.** Black dashed lines showed approximate tumor margins; black dashed squares showed the magnified regions. Scale bar, 500 μm or 250 μm (amplification). **(B)** Representative immunofluorescent staining of activated DCs in the experimental mouse model. White triangles indicated the cells shown in the upper-left insets in each image. *n* = 6 mice per group. Scale bar, 200 μm. **(C-E)** The DCs were isolated from the lesional and non-lesional skin tissues of the mice bearing breast cancer, applied to *in vitro* function assays and analyzed by flow cytometry or spectrophotometry. *n* = 6 mice per group. **(C)** Representative flow cytometry plots showed the expression of MHC-II, CD80 and CD86, as well as the uptake and processing of fluorescence-conjugated OVA and bioparticles by DCs. **(D)** Representative flow cytometry plots showed the proliferation and expression of cytotoxic cytokines in T lymphocytes primed by DCs. **(E)** The cytotoxic effect of T lymphocytes primed by DCs on tumor cells was assessed using LDH release assay. Data were mean ± SD; significance was determined using a two-tailed Student's *t*-test (**B**; CFSE in **D**; **E**) or Mann-Whitney's *U*-test (others in **C** and **D**). CD, cluster of differentiation; CFSE, 5(6)-carboxyfluorescein diacetate N-succinimidyl

ester; Fluor, fluorescence; IFN, interferon; LDH, lactate dehydrogenase; MFI, mean fluorescence intensity; MHC, major histocompatibility complex; non-SI, mice bearing breast cancer without skin involvement; OVA, ovalbumin; SI, mice bearing breast cancer with skin involvement.

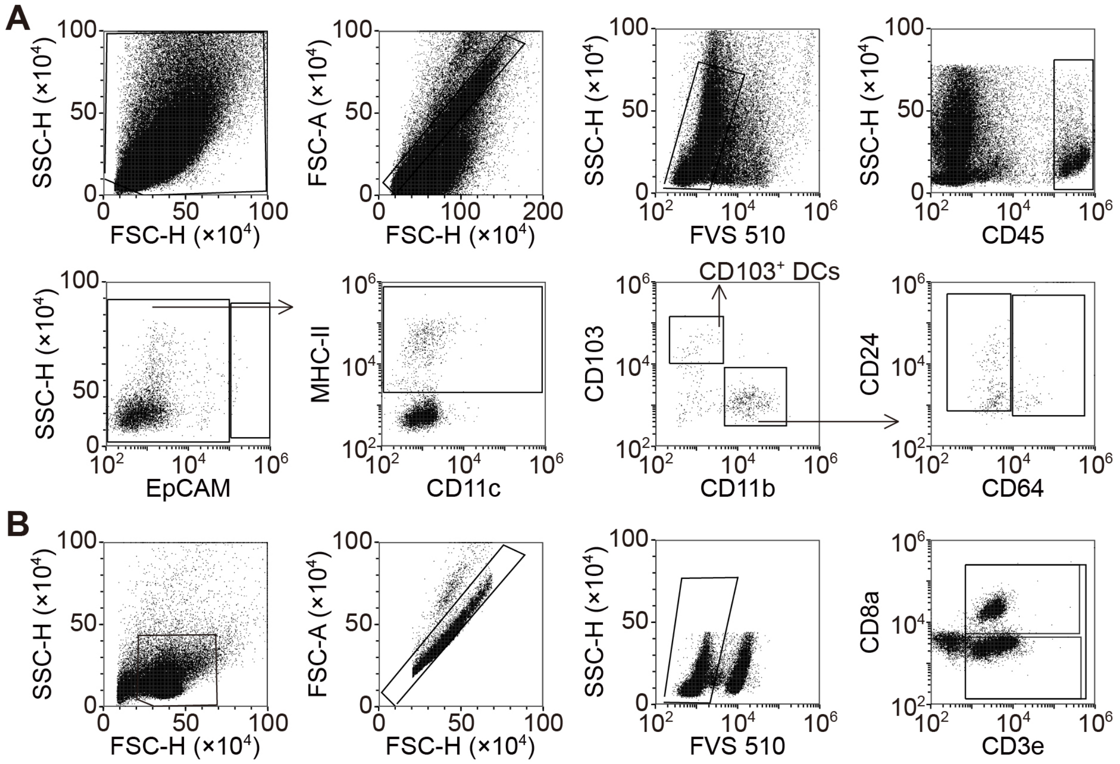

Figure S3 Flow cytometry gating strategies for mouse samples.

(A) The DCs were isolated from the lesional and non-lesional skin tissues of mice bearing breast cancer, applied to *in vitro* function assays, harvested and stained for specific markers in the presence of anti-mouse CD16/CD32 antibody (Mouse BD Fc Block) and analyzed by flow cytometry. Representative flow cytometry plots showed a typical gating strategy to identify DCs. Single viable $CD45^+$ immune cells were first identified as follows: forward scatter (FSC) and side scatter (SSC) to identify most cells and exclude debris; FSC height-area ratio to exclude doublets; Fixable viability stain $(FVS)^-$ to identify viable cells; and subsequent $CD45^+$ to identify leukocytes. Single viable $CD45^+$ immune cells were further gated to identify MHC-II^+ cells and $CD103^+$MHC-II^+ DCs were then identified as follows: $EpCAM^-$ to exclude Langerhans cells (LCs); CD11c and MHC-II to identify conventional DCs; CD103 and CD11b to identify $CD103^+$ DCs. Furthermore, optionally, within the last gate cells could be identified as $CD24^+CD11b^+$ DCs and $CD64^+$ macrophages. **(B)** The DCs were isolated from the lesional and non-lesional skin tissues of the mice bearing breast cancer and applied to *in vitro* mixed leukocyte reaction (MLR, T lymphocyte priming) assays;

suspended T lymphocytes were harvested, stained for specific markers and analyzed by flow cytometry. Representative flow cytometry plots showed a typical gating strategy to identify T lymphocytes. Single viable cells were first identified as (**A**) described, but no CD45 gating was previously used because of aforehand processes of T lymphocytes isolation. T lymphocytes were further identified as follows: $CD3e^{+}$ to identify total T lymphocytes (for proliferation analyses); $CD8a^{+}$ to identify cytotoxic T lymphocytes among them (for cytotoxic cytokines expression analyses). CD, cluster of differentiation; DC, dendritic cell; EpCAM, epidermal cell adhesion molecule, also known as CD326; FSC, forward scatter; FVS, Fixable viability stain; MHC, major histocompatibility complex; SSC, side scatter.

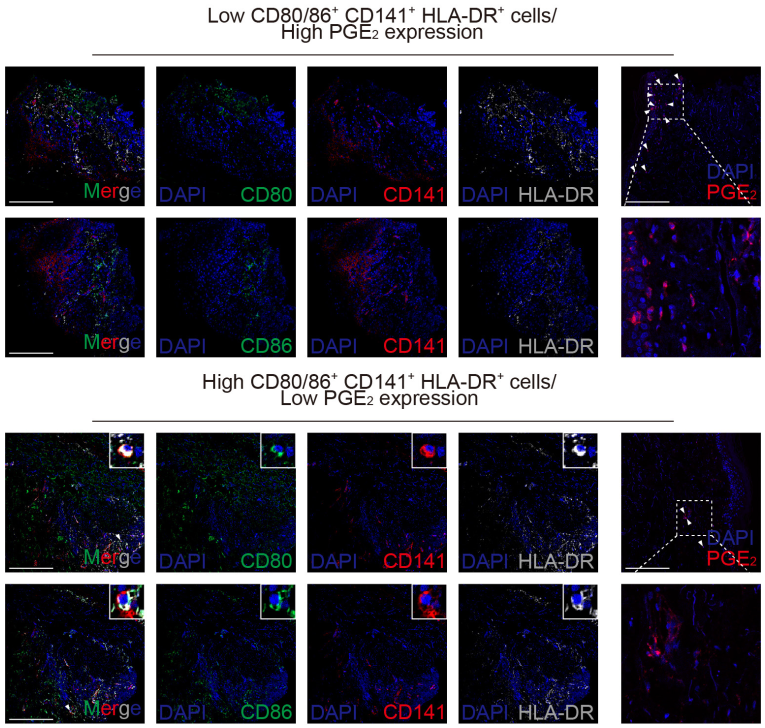

Figure S4 PGE$_2$ expression and activated DCs in two typical breast cancer patients. Representative immunofluorescent staining of activated DCs (left panel) and PGE$_2$ (right panel) in skin tissues of the two typical breast cancer patients with skin involvement with opposite activated DC infiltration and PGE$_2$ expression situations showing different clinical outcomes as illustrated in Figure 4F. White triangles indicated the cells shown in the upper-right insets in each image (left panel) or PGE$_2$ (right panel); white dashed squares showed the magnified regions. Scale bar, 200 μm. CD, cluster of differentiation; DAPI, 4',6-diamidino-2-phenylindole; HLA, human leukocyte antigen; PGE$_2$, prostaglandin E$_2$.

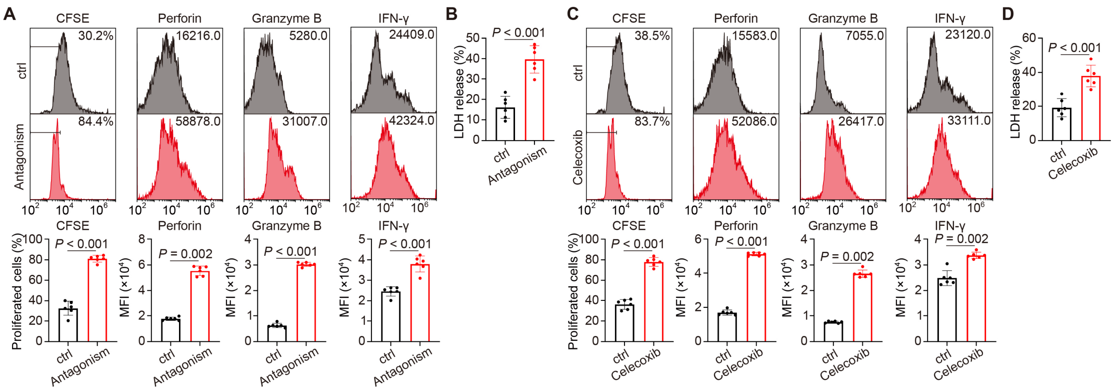

Figure S5 PGE_2 inhibition restored DC priming function in the mouse model.

(A, B) The breast cancer mouse model with skin involvement was treated with either the control or PGE_2 antagonism. *n* = 6 mice per group (**A**) or 3 biological replicates per group (**B**). Cutaneous DCs isolated from mice bearing breast cancer that received either the control or PGE_2 antagonism treatment were subjected to *in vitro* function assays and analyzed by flow cytometry or spectrophotometry. **(A)** Representative flow cytometry plots showed the proliferation and expression of cytotoxic cytokines in T lymphocytes primed by DCs. **(B)** The cytotoxic effect of T lymphocytes primed by DCs on tumor cells was assessed using LDH release assay. **(C, D)** The breast cancer mouse model with skin involvement was treated with either the control or celecoxib. *n* = 6 mice per group (**C**) or 3 biological replicates per group (**D**). Cutaneous DCs isolated from mice bearing breast cancer that received either the control or celecoxib were subjected to *in vitro* function assays and analyzed by flow cytometry or spectrophotometry. **(C)** Representative flow cytometry plots showed the proliferation and expression of cytotoxic cytokines in T lymphocytes primed by DCs. **(D)** The cytotoxic effect of T lymphocytes primed by DCs on tumor cells was assessed using LDH release assay. Data were mean ± SD; significance was determined using a two-tailed Student's *t*-test (CFSE, Granzyme B and IFN-γ in **A**; **B**; CFSE and Perforin in **C**; **D**) or Mann-Whitney's *U*-test (others in **A** and **C**). CFSE, 5(6)-carboxyfluorescein diacetate N-succinimidyl ester; ctrl, control; IFN, interferon; LDH, lactate dehydrogenase; MFI, mean fluorescence intensity.

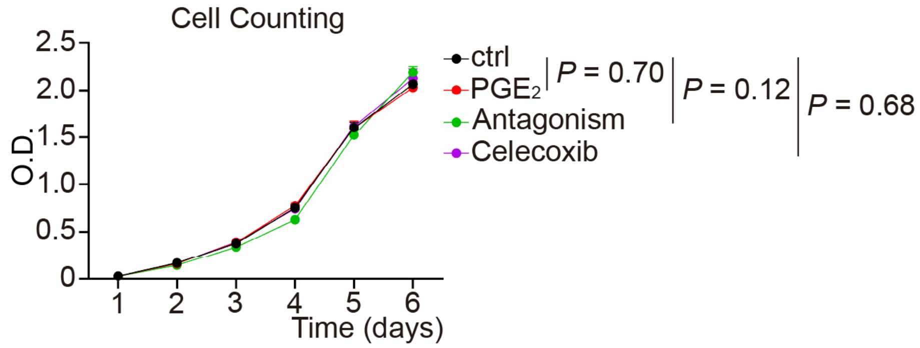

Figure S6 PGE_2 or its inhibition exerted negligible influence on cell proliferation. Cell proliferation of EO771 breast cancer cells receiving the control, PGE_2, PGE_2 antagonism or celecoxib treatment was measured by CCK-8. Data were mean ± SD; significance was determined using a two-tailed two-way analysis of variance (ANOVA) and subsequent Dunnett's multiple comparisons test. ctrl, control; O.D., optical density; PGE_2, prostaglandin E_2.

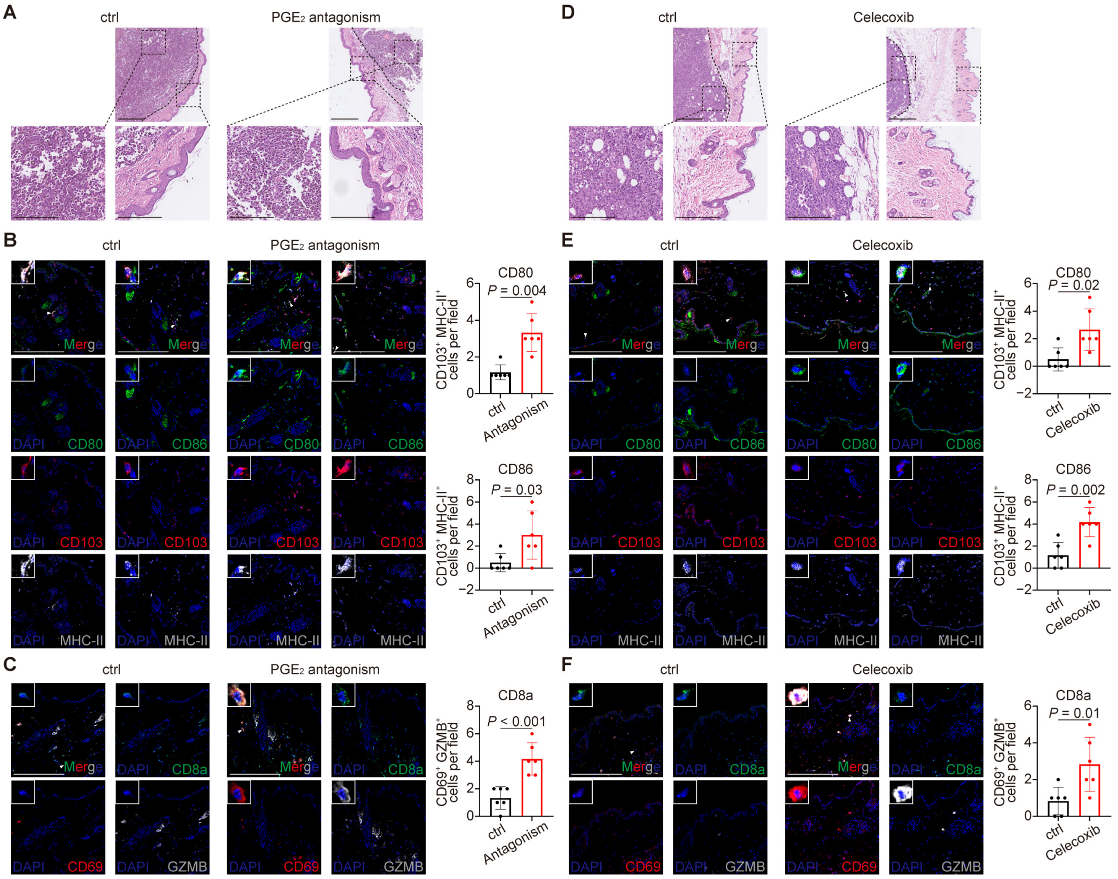

Figure S7 PGE_2 inhibition relieved skin involvement and restored DC activation in the melanoma mouse model.

(A-C) The melanoma mouse model with skin involvement was treated with either the control or PGE_2 antagonism. *n* = 6 mice per group. **(A)** Representative H&E images depicting skin involvement in the melanoma mouse model. Black dashed lines showed approximate tumor margins; black dashed squares showed the magnified regions. Scale bar, 500 μm or 250 μm (amplification). **(B, C)** Representative immunofluorescent staining of activated DCs (**B**) and functional T lymphocytes (**C**) in skin tissues from the melanoma mouse model. White triangles indicated the cells shown in the upper-left insets in each image. Scale bar, 200 μm. **(D-F)** The melanoma mouse model with skin involvement was treated with either the control or celecoxib. *n* = 6 mice per group. **(D)** Representative H&E images depicting skin involvement in the melanoma mouse model. Black dashed lines showed approximate tumor margins; black dashed squares showed the magnified regions. Scale bar, 500 μm or 250 μm (amplification). **(E, F)**

Representative immunofluorescent staining of activated DCs (**E**) and functional T lymphocytes (**F**) in skin tissues from the melanoma mouse model. White triangles indicated the cells shown in the upper-left insets in each image. Scale bar, 200 μm. Data were mean ± SD; significance was determined using a two-tailed Student's *t*-test (CD86 in **B**; **C**; CD86 in **E**; **F**) or Mann-Whitney's *U*-test (others in **B** and **E**). CD, cluster of differentiation; ctrl, control; DAPI, 4',6-diamidino-2-phenylindole; GZMB, granzyme B; MHC, major histocompatibility complex; PGE_2, prostaglandin E_2.

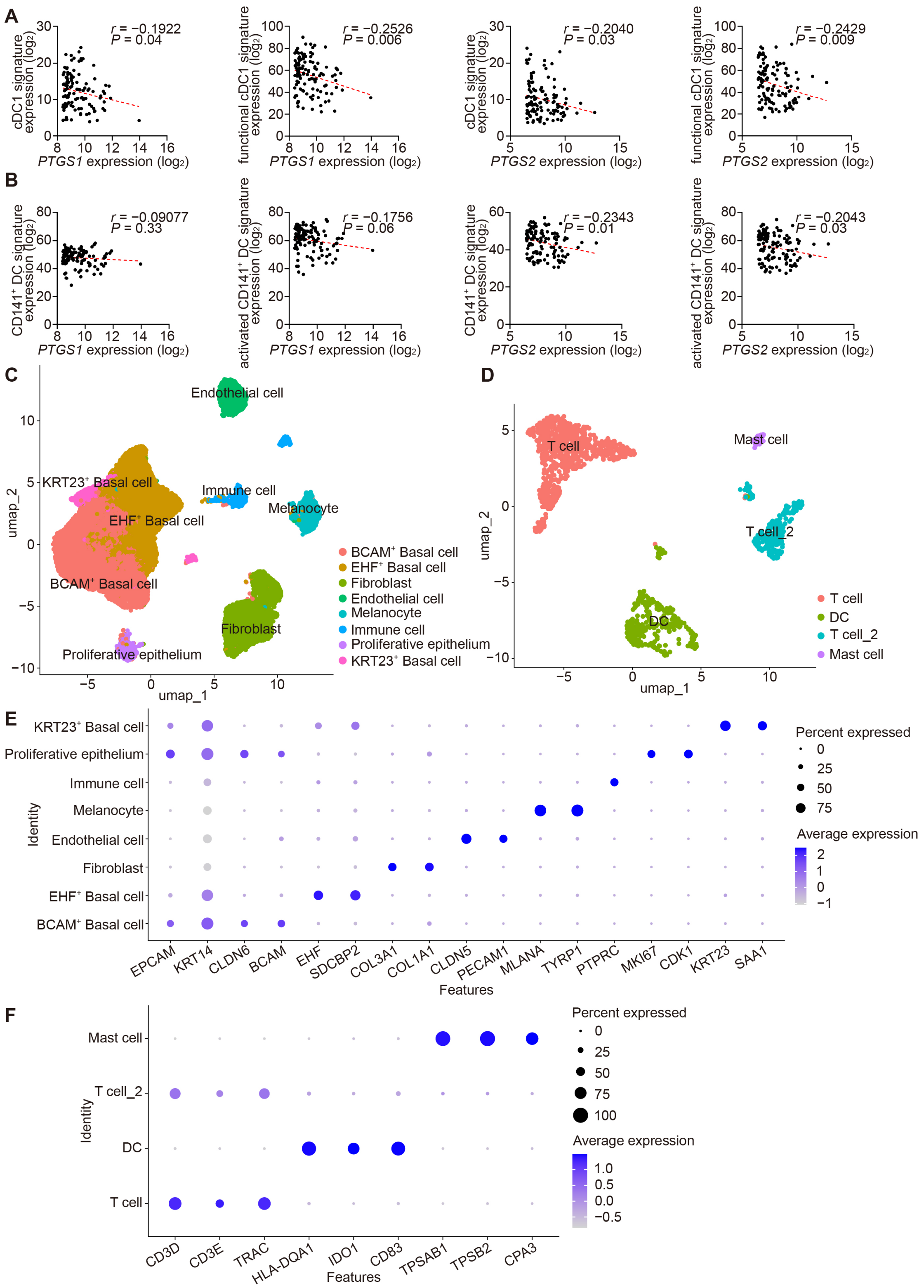

Figure S8 Bioinformatic re-analyses of sequencing datasets of skin cutaneous melanoma and basal cell carcinoma patients.

(A, B) Re-analyzing the dataset from The Cancer Genome Atlas (TCGA) skin cutaneous melanoma (SKCM). **(A)** The scatter plots showed the correlation between

PTGS1 or *PTGS2* expression levels and cDC1 or functional cDC1 signature gene expression. *n* = 115; each dot represented one sample. A linear regression-fitting curve was shown as a red dashed line. **(B)** The scatter plots showed the correlation between *PTGS1* or *PTGS2* expression levels and CD141$^+$ DC or activated CD141$^+$ DC signature gene expression. *n* = 115; each dot represented one sample. A linear regression-fitting curve was shown as a red dashed line. **(C-F)** Re-analyzing the publicly available single-cell sequencing dataset of basal cell carcinoma (BCC) patients (GSE141526). **(C, D)** UMAP plots showed different clusters of total cells (**C**) and immune cells therein (**D**) in lesional and non-lesional skin of BCC patients. **(E, F)** Dot plots showed differentially expressed marker genes of total cells (**E**) or immune cells therein (**F**) in lesional and non-lesional skin of BCC patients. Significance was determined using Pearson's correlation coefficient (*r*) and subsequent *t*-test. CD, cluster of differentiation; DC, dendritic cell; PTGS, prostaglandin-endoperoxide synthase.

Supplementary references

1. Haniffa M, Shin A, Bigley V, McGovern N, Teo P, See P, et al. Human tissues contain CD141hi cross-presenting dendritic cells with functional homology to mouse CD103+ nonlymphoid dendritic cells. Immunity. 2012;37(1):60-73.

2. Qian H, Leng X, Wen J, Zhou Q, Xu X, Wu X. One-Step Simple Isolation Method to Obtain Both Epidermal and Dermal Stem Cells from Human Skin Specimen. Methods in molecular biology (Clifton, NJ). 2019;1879:139-48.

3. Vieira Braga FA, Miragaia RJ. Tissue Handling and Dissociation for Single-Cell RNA-Seq. Methods in molecular biology (Clifton, NJ). 2019;1979:9-21.

4. Liu J, Lao L, Chen J, Li J, Zeng W, Zhu X, et al. The IRENA lncRNA converts chemotherapy-polarized tumor-suppressing macrophages to tumor-promoting phenotypes in breast cancer. Nat Cancer. 2021;2(4):457-73.

5. Zhang W, Wang J, Liang J, He Z, Wang K, Lin H. RNA methylation of CD47 mediates tumor immunosuppression in EGFR-TKI resistant NSCLC. British Journal of Cancer. 2025.

6. Yang L, Liu Q, Zhang X, Liu X, Zhou B, Chen J, et al. DNA of neutrophil extracellular traps promotes cancer metastasis via CCDC25. Nature. 2020;583(7814):133-8.

7. Zelenay S, van der Veen AG, Bottcher JP, Snelgrove KJ, Rogers N, Acton SE, et al. Cyclooxygenase-Dependent Tumor Growth through Evasion of Immunity. Cell. 2015;162(6):1257-70.

8. Breton G, Zheng S, Valieris R, Tojal da Silva I, Satija R, Nussenzweig MC. Human dendritic cells (DCs) are derived from distinct circulating precursors that are precommitted to become CD1c+ or CD141+ DCs. The Journal of experimental medicine. 2016;213(13):2861-70.

9. Cossarizza A, Chang HD, Radbruch A, Acs A, Adam D, Adam-Klages S, et al. Guidelines for the use of flow cytometry and cell sorting in immunological studies (second edition). European journal of immunology. 2019;49(10):1457-973.

10. Kramer K, Young SL, Walker GF. Data on the uptake of reducible antigen-adjuvant conjugates by dendritic cells. Data Brief. 2019;23:103759.

11. Lindner B, Burkard T, Schuler M. Phagocytosis assays with different pH-sensitive fluorescent particles and various readouts. Biotechniques. 2020;68(5):245-50.

12. Su S, Zhao J, Xing Y, Zhang X, Liu J, Ouyang Q, et al. Immune Checkpoint Inhibition Overcomes ADCP-Induced Immunosuppression by Macrophages. Cell. 2018;175(2):442-57.e23.

13. Steinman RM, Gutchinov B, Witmer MD, Nussenzweig MC. Dendritic cells are the principal stimulators of the primary mixed leukocyte reaction in mice. The Journal of experimental medicine. 1983;157(2):613-27.

14. Bayerl F, Meiser P, Donakonda S, Hirschberger A, Lacher SB, Pedde AM, et al. Tumor-derived prostaglandin E2 programs cDC1 dysfunction to impair intratumoral orchestration of anti-cancer T cell responses. Immunity. 2023;56(6):1341-58 e11.

15. Zhang JH, Miao NY, Lao LY, Deng W, Wang JW, Zhu XF, et al. Activation of Bivalent Gene POU4F1 Promotes and Maintains Basal-like Breast Cancer. Advanced Science. 2024;11(20):e2307660.

16. Huang D, Zhu X, Ye S, Zhang J, Liao J, Zhang N, et al. Tumour circular RNAs elicit anti-tumour immunity by encoding cryptic peptides. Nature. 2024;625(7995):593-602.

17. Tvingsholm SA, Frej MS, Rafa VM, Hansen UK, Ormhøj M, Tyron A, et al. TCR-engaging scaffolds selectively expand antigen-specific T-cells with a favorable phenotype for adoptive cell therapy. Journal for immunotherapy of cancer. 2023;11(8).

18. Lee PJ, Sui YH, Liu TT, Tsang NM, Huang CH, Lin TY, et al. Epstein-Barr viral product-containing exosomes promote fibrosis and nasopharyngeal carcinoma progression through activation of YAP1/FAPalpha signaling in fibroblasts. Journal of experimental & clinical cancer research : CR. 2022;41(1):254.

19. Kim D, Langmead B, Salzberg SL. HISAT: a fast spliced aligner with low memory requirements. Nat Methods. 2015;12(4):357-60.

20. Yao CD, Haensel D, Gaddam S, Patel T, Atwood SX, Sarin KY, et al. AP-1 and TGFss cooperativity drives non-canonical Hedgehog signaling in resistant basal cell carcinoma. Nat Commun. 2020;11(1):5079.

21. Guerrero-Juarez CF, Lee GH, Liu Y, Wang S, Karikomi M, Sha Y, et al. Single-cell analysis of human basal cell carcinoma reveals novel regulators of tumor growth and the tumor microenvironment. Science advances. 2022;8(23):eabm7981.

22. Hao Y, Stuart T, Kowalski MH, Choudhary S, Hoffman P, Hartman A, et al. Dictionary learning for integrative, multimodal and scalable single-cell analysis. Nat Biotechnol. 2024;42(2):293-304.

23. Pang Z, Lu Y, Zhou G, Hui F, Xu L, Viau C, et al. MetaboAnalyst 6.0: towards a unified platform for metabolomics data processing, analysis and interpretation. Nucleic Acids Research. 2024;52(W1):W398-W406.

24. Bottcher JP, Bonavita E, Chakravarty P, Blees H, Cabeza-Cabrerizo M, Sammicheli S, et al. NK Cells Stimulate Recruitment of cDC1 into the Tumor Microenvironment Promoting Cancer Immune Control. Cell. 2018;172(5):1022-37 e14.

25. Jiang P, Gu S, Pan D, Fu J, Sahu A, Hu X, et al. Signatures of T cell dysfunction and exclusion predict cancer immunotherapy response. Nature Medicine. 2018;24(10):1550-8.

26. Cooles FAH, Isaacs JD. The interferon gene signature as a clinically relevant biomarker in autoimmune rheumatic disease. The Lancet Rheumatology. 2022;4(1):e61-e72.
